# *circPTPN4* regulates myogenesis via the *miR-499-3p*/*NAMPT* axis

**DOI:** 10.1186/s40104-021-00664-1

**Published:** 2022-02-14

**Authors:** Bolin Cai, Manting Ma, Zhen Zhou, Shaofen Kong, Jing Zhang, Xiquan Zhang, Qinghua Nie

**Affiliations:** 1 State Key Laboratory for Conservation and Utilization of Subtropical Agro-Bioresources, Lingnan Guangdong Laboratory of Agriculture, College of Animal Science, South China Agricultural University, Guangzhou, 510642 Guangdong China; 2 Guangdong Provincial Key Lab of Agro-Animal Genomics and Molecular Breeding, and Key Laboratory of Chicken Genetics, Breeding and Reproduction, Ministry of Agriculture, Guangzhou, 510642 Guangdong China

**Keywords:** Chicken, *CircPTPN4*, Circular RNA, *MiR-499-3p*, Myogenesis, *NAMPT*, The transformation of myofiber

## Abstract

**Background:**

Circular RNAs (circRNAs) are a novel class of endogenous ncRNA, which widely exist in the transcriptomes of different species and tissues. Recent studies indicate important roles for circRNAs in the regulation of gene expression by acting as competing endogenous RNAs (ceRNAs). However, the specific role of circRNAs in myogenesis is still poorly understood. In this study, we attempted to systematically identify the circRNAs involved in myogenesis and analyze the biological functions of circRNAs in chicken skeletal muscle development.

**Results:**

In total, 532 circRNAs were identified as being differentially expressed between pectoralis major (PEM) and soleus (SOL) in 7-week-old Xinghua chicken. Among them, a novel circRNA (novel_circ_002621), generated by *PTPN4* gene, was named *circPTPN4* and identified. *circPTPN4* is highly expressed in skeletal muscle, and its expression level is upregulated during myoblast differentiation. *circPTPN4* facilitates the proliferation and differentiation of myoblast. Moreover, *circPTPN4* suppresses mitochondria biogenesis and activates fast-twitch muscle phenotype. Mechanistically, *circPTPN4* can function as a ceRNA to regulate *NAMPT* expression by sponging *miR-499-3p*, thus participating in AMPK signaling.

**Conclusions:**

*circPTPN4* functions as a ceRNA to regulate *NAMPT* expression by sponging *miR-499-3p*, thus promoting the proliferation and differentiation of myoblast, as well as activating fast-twitch muscle phenotype.

**Supplementary Information:**

The online version contains supplementary material available at 10.1186/s40104-021-00664-1.

## Background

Chicken is the second most consumed meat in China, and the meat production performance of chicken determines its commercial value. While increasing the yield, improving the quality of chicken is the direction that poultry breeders have been working hard on. Recently, it has come to light that the composition of myofiber types has an important relationship with muscle quality [1, 2]. The discovery of genetic regulatory factors involved in skeletal muscle development is of great significance to chicken production.

Gene is the carrier of genetic information, carrying various biological processes of life. The product, such as peptide or protein molecules, plays a key role in it [3]. However, protein-encoding genes only account for a small portion (~ 2%) of the genome, while more than 98% of the genomic loci are transcribed to noncoding RNAs (ncRNAs) [4]. Skeletal muscle is the largest tissue in the body, which comprises about 40% of the total body mass. The development of skeletal muscle is closely related to growth and health, and is directly regulated by multiple genetic factors. Noticeably, recent studies have found that ncRNAs play critical roles in it [5, 6].

Circular RNAs (circRNAs) are a novel class of endogenous ncRNA with a covalently closed loop, which widely exist in the transcriptomes of different species and tissues [7, 8]. Compared with linear RNA (such as long noncoding RNA), circRNA has higher structural stability and conservation. It is becoming increasingly clear that circRNAs can widely be involved in a series of biological processes by acting as a miRNA sponge, participating in regulating the expression of its own linear RNA in different ways, coding protein, and deriving pseudogenes [9–12]. Although, more and more circRNAs have been found by high-throughput sequencing, the mechanism of circRNA regulation involved in skeletal muscle development is still poorly understood.

MicroRNAs (miRNAs) are endogenous noncoding single-stranded RNA molecules of 18–22 nt long that are capable of degrading or inhibiting target mRNAs by perfect or imperfect pairing with the 3′ untranslated region (3′ UTR) of the target mRNA to regulate post-transcriptional gene expression [13, 14]. Recent study has found that *miR-499-3p* could suppress retinal cell proliferation while promote apoptosis to induce diabetic retinopathy by enhancing activation of the TLR4 signaling pathway [15]. In pigs, the expression of *ssc-miR-499-3p* was significantly correlated to the expression of myoglobin and pH, prompting its potential regulatory role in skeletal muscle fiber transformation and meat quality traits [16]. However, the exact biological function of *miR-499-3p* in skeletal muscle development has not been reported yet.

Nicotinamide phosphoribosyltransferase (*NAMPT*) is the rate-limiting enzyme which catalyzes the conversion of nicotinamide and phosphoribosyl-pyrophosphates to nicotinamide mononucleotide in the mammalian nicotinamide adenine dinucleotide (NAD^+^) synthetic salvage pathway [17, 18]. Recently, numerous studies have indicated that *NAMPT* is able to modulate processes involved in the pathogenesis of obesity and related disorders by influencing the oxidative stress response, apoptosis, lipid and glucose metabolism, inflammation and insulin resistance [19]. But little is known about how *NAMPT* functions in skeletal muscle development.

In this study, to systematically identify the circRNAs involved in skeletal muscle development, pectoralis major (PEM) and soleus (SOL) in 7-week-old Xinghua chicken were used for circRNA sequencing (circRNA-seq). Based on this result, a novel circRNA (novel_circ_002621), generated by the *PTPN4* gene, was identified and named *circPTPN4*. *circPTPN4* is highly expressed in skeletal muscle, and its expression upregulates with myoblast differentiation. Functional studies demonstrated that *circPTPN4* promotes the proliferation and differentiation of myoblast, as well as activates the fast-twitch muscle phenotype. Furthermore, the mechanistic investigation revealed that *circPTPN4* can function as a competing endogenous RNA (ceRNA) by sponging *miR-499-3p*, thus regulating the expression of *NAMPT* to mediate the AMPK signaling.

## Methods

### Ethics statement

All animal experimental protocols were conformed to “The Instructive Notions with Respect to Caring for Laboratory Animals” issued by the Ministry of Science and Technology of the People’s Republic of China, and approved by the Institutional Animal Care and Use Committee at the South China Agricultural University (approval ID: 2021-C018).

### Animals and cells

Seven-week-old Xinghua female chickens were hatched from the Avian Farm of South China Agricultural University (Guangzhou, China). The chickens were euthanized, and organs and tissues were collected after rapid dissection, then immediately frozen in liquid nitrogen and stored at − 80 °C.

Chicken primary myoblasts (CPMs) were isolated from leg muscles of E11 (11-embryonic-day-old) chicken and cultured as previously described [20]. Firstly, the muscle tissues were dissected away from the skin and bone, and then homogenized in a petri dish. To release single cells, the suspension was digested with pancreatin for 20 min at 37 °C. After neutralization with complete medium, single cells were collected by centrifugation at 500 × *g*. Subsequently, serial plating was performed to enrich primary myoblasts and eliminate fibroblasts. Primary myoblasts were cultured in Roswell Park Memorial Institute (RPMI)-1640 medium (Gibco, MD, USA) with 20% FBS, 1% nonessential amino acids, and 0.2% penicillin/streptomycin. The purity of isolated primary myoblasts was verified by immunofluorescence (Fig. S1).

To induce myogenic differentiation, the growth medium was removed and replaced with differentiation medium (RPMI-1640 medium [Gibco, MD, USA] containing 2% horse serum) after myoblasts achieved 90% cell confluence.

### Circular RNA sequencing (circRNA-seq)

The pectoralis major (PEM; which is mainly composed of fast-twitch fibers) and soleus (SOL; which has higher proportion of slow muscle fibers) of 7-week-old Xinghua chicken were used for circRNA-seq. After extraction, total RNAs were treated with RNase R to degrade the linear RNAs, and purified using RNeasy MinElute Cleanup Kit (Qiagen, Walldorf, Germany). Next, strand-specific library was constructed using VAHTS Total RNA-seq (H/M/R) Library Prep Kit for Illumina following the manufacturer’s instructions. Briefly, ribosome RNAs were removed to retain circRNAs. The enriched circRNAs were fragmented into short fragments by using fragmentation buffer and reverse transcripted into cDNA with random primers. Second-strand cDNA were synthesized by DNA polymerase I, RNase H, dNTP (dUTP instead of dTTP) and buffer. Next, the cDNA fragments were purified with VAHTSTM DNA Clean Beads, end repaired, poly(A) added, and ligated to Illumina sequencing adapters. Then UNG (Uracil-N-Glycosylase) was used to digest the second-strand cDNA. The digested products were purified with VAHTSTM DNA Clean Beads, PCR amplified, and sequenced using Illumina HiSeq™ 2500 by Gene Denovo Biotechnology Co. (Guangzhou, China). The raw data of circRNA-seq were deposited in the Sequence Read Archive (SRA) database under accession no. PRJNA751251.

Parental genes of differentially expressed circRNAs were subjected to enrichment analysis of Gene Ontology (GO) functions and Kyoto Encyclopedia of Genes and Genomes (KEGG) pathways.

### Validation of circRNA

The circRNAs were validated using PCR with divergent and convergent primers as previously described [21]. To confirm the junction sequence of circRNAs, PCR products of divergent primers were gel purified and submitted for Sanger sequencing at Tsingke Biotechnology Co., Ltd. (Beijing, China). To check the sensitivity of circRNA to RNase R, quantitative PCR was also performed using RNA samples with and without RNase R treatment. Primers used for the validation of circRNA are summarized in Table S1.

### RNA extraction, cDNA synthesis, and quantitative real-time PCR

Total RNA was extracted by using the TRIzol reagent (TaKaRa, Otsu, Japan), following the manufacturer’s protocol. Nuclear and cytoplasmic RNA fractionation was performed by using the Paris kit (Ambion, Life Technologies, Carlsbad, CA, USA) as recommended by the supplier. The PrimeScript RT Reagent Kit with gDNA Eraser (Perfect Real Time) (TaKaRa, Otsu, Japan) was used to synthesize cDNA. Quantitative real-time PCR was performed as described before [22]. And primers used for quantitative real-time PCR are listed in Table S1.

### Plasmid construction and RNA oligonucleotides

For pGL3 luciferase reporter vectors construction, the active region of *PTPN4* gene promoter containing FOXA2 binding site and FOXA2 binding site mutant were amplified and cloned into the pGL3-Basic Vector (Promega, Madison, WI, USA) by using *XhoI* and *HindIII* restriction sites.

For *FOXA2* expression vectors construction, the coding sequence of *FOXA2* was amplified by PCR, and then subcloned into *HindIII* and *XhoI* restriction sites of the pcDNA3.1-3xFLAG-C vector or cloned into the expression plasmid pcDNA-3.1 (Promega, Madison, WI, USA) by using *HindIII* and *XhoI* restriction sites.

For *circPTPN4* overexpression vector construction, the linear sequence of *circPTPN4* was amplified and then subcloned into *EcoRI* and *BamHI* restriction sites of the pCD25-ciR vector (Geneseed Biotech, Guangzhou, China) by using the Trelief™ SoSoo Cloning Kit (Tsingke Biotech, Beijing, China), following the manufacturer’s protocol.

For pmirGLO dual-luciferase miRNA target reporter vector, the segment sequences of *circPTPN4* and *NAMPT* 3′ untranslated region (UTR) that contained the putative *miR-499-3p* binding sequence were amplified by PCR, and then subcloned into *XhoI* and *SalI* restriction sites in the pmirGLO dual luciferase reporter vector (Promega, Madison, WI, USA). Mutant plasmids were generated by changing the binding site of *miR-499-3p* from GTGATGT to TGTCGTG.

*miR-499-3p* mimic, mimic negative control (NC), 3′ end biotinylated *miR-499-3p* mimic, 3′ end biotinylated mimic NC and small interfering RNA (siRNA) against *circPTPN4* were designed and synthesized by Guangzhou RiboBio (Guangzhou, China).

The primers and oligonucleotide sequences used in this study are listed in Tables S1 and S2.

### Cell transfection

All transient transfections were performed with Lipofectamine 3000 reagent (Invitrogen, Carlsbad, CA, USA) according to manufacturer’s directions.

### Dual-luciferase reporter assay

Dual-luciferase reporter assays were performed as previously described [23, 24]. For promoter activity assay, the pGL3-basic vectors were co-transfected with pRL-TK as a control. Firefly and Renilla luciferase activities were measured at 48 h post-transfection using a Dual-GLO Luciferase Assay System Kit (Promega, Madison, WI, USA), following the manufacturer’s instructions. Luminescence was measured by using a Fluorescence/Multi-Detection Microplate Reader (BioTek, Winooski, VT, USA) and firefly luciferase activities were normalized to Renilla luminescence in each well.

### Chromatin immunoprecipitation (ChIP) assay

ChIP assay was performed by using the ChIP assay kit (Beyotime, Shanghai, China) as recommended by the supplier. Chromatin was immunoprecipitated with the DYKDDDDK Tag (D6W5B) rabbit monoclonal antibody (14,793, 1:50, Cell Signaling Technology, Inc., Boston, USA). The relative quantity of the immunoprecipitated factor was calculated by qPCR.

### 5-Ethynyl-2′-deoxyuridine (EdU), flow cytometry, and cell counting kit-8 (CCK-8) assay

For the EdU assay, primary myoblasts seeded in 24-well plates were cultured to 50% density and then transfected. Forty-eight hours after transfection, the cells were fixed and stained with a C10310 EdU Apollo In Vitro Imaging Kit (RiboBio, China; 50 μmol/L) as previously described [23]. A fluorescence microscope (DMi8; Leica, German) was used to capture three randomly selected fields to visualize the number of EdU-stained cells.

For the flow cytometry analysis of the cell cycle, myoblasts were seeded in 12-well plates. After 48 h transfection, the cultured cells in growth media were collected and fixed overnight in 70% ethanol at − 20 °C. Cells were analyzed by a BD AccuriC6 flow cytometer (BD Biosciences, San Jose, CA, USA) with the Cell Cycle Analysis Kit (Thermo Fisher Scientific, USA), and the data were processed using FlowJo software (7.6, Treestar Incorporated, Ashland, OR, USA).

For the CCK-8 assay, primary myoblasts were seeded in a 96-well plate and cultured in growth medium. After being transfected, the proliferation of the cell culture was monitored at 12 h, 24 h, 36 h, and 48 h using the TransDetect CCK (TransGen Biotech, Beijing, China) as recommended by the supplier. The data of absorbance at 450 nm were read by an iMark™ Microplate Absorbance Reader (Bio-Rad, California, USA).

### Immunoblotting and immunofluorescence (IF)

Western blots were performed as previously described [20]. The primary antibodies used were anti-MyHC (B103, 0.5 μg/mL, DHSB, Iowa City, IA, USA), anti-MYOD (ABP53067, 1:500, Abbkine, Wuhan, China), anti-MYH1A (F59, 0.5 μg/mL, DHSB, Iowa City, IA, USA), anti-MYH7B (S58, 0.5 μg/mL, DHSB, Iowa City, IA, USA), anti-NAMPT (bs-0272R, 1:500, Bioss, Beijing, China), anti-p-AMPK (ABN-PAB12602, 1:2000, Abnova, Taipei City, Taiwan, China), anti-AMPK (bs-1115R, 1:500, Bioss, Beijing, China), anti-PGC1α (66369–1-Ig, 1:5000, Proteintech, IL, USA), and anti-β-Tubulin (A01030, 1:10,000, Abbkine, Wuhan, China). ProteinFind Goat Anti-Mouse IgG (H + L), HRP Conjugate (HS201–01, 1:1000, TransGen, Beijing, China) and ProteinFind Goat Anti-Rabbit IgG (H + L), HRP Conjugate (HS101–01, 1:500, TransGen, Beijing, China) were used as a secondary antibody.

Immunofluorescence were performed using anti-Desmin (bs-1026R, 1:100, Bioss, Beijing, China) and anti-MyHC (B103, 2.5 μg/mL, DHSB, Iowa City, IA, USA), as previously described [20]. A fluorescence microscope (DMi8; Leica, Germany) was used to capture three randomly selected fields to visualize the area labeled with anti-MyHC.

### Mitochondrial DNA (mtDNA) content assay

Total DNA was extracted by using the Tissue DNA Kit (D3396, Omega, GA, USA) according to the manufacturer’s instructions. The amount of mitochondrial DNA was determined by quantification of cytochrome c oxidase subunit II (*COX2*). The nuclear-encoded *β*-globin gene was used as internal controls. Primers used in this study can be found in the Table S1.

### Mitochondrial membrane potential and reactive oxygen species (ROS) concentration assay

Mitochondrial membrane potential and ROS concentration were measured using the mitochondrial membrane potential assay kit with JC-1 (C2006, Beyotime, Shanghai, China) and reactive oxygen species assay kit (S0033S, Beyotime, Shanghai, China), according to the manufacturer’s instructions.

### Enzyme activities assays

The glycolytic capacity of myoblast was evaluated by the activity of lactic dehydrogenase (LDH), while the oxidative capacity of myoblast was evaluated by the activity of succinate dehydrogenase (SDH). Enzyme activities were measured by commercial assay kits (BC0685 and BC0955) that were purchased from Beijing Solarbio Science & Technology.

### Biotin-coupled miRNA pull down assay

The 3′ end biotinylated *miR-499-3p* mimic and mimic NC were transfected into CPMs in T75 cell culture bottle. At 48 h after transfection, the cells were harvested and then lysed in lysis buffer. The biotin-coupled RNA complex was pull down, and then isolated as previously described [25]. The abundance of *circPTPN4* and *NAMPT* in bound fractions was evaluated by quantitative PCR.

### Statistical analysis

In this study, all experiments were repeated at least three times, and results were represented as mean ± SEM. Where applicable, the statistical significance of the data was tested using independent sample *t*-test or ANOVA followed by Dunnett’s test. The types of tests and the *P*-values, when applicable, are indicated in the figure legends.

## Results

### Characterization of circRNAs in fast-twitch and slow-twitch myofiber

**Fig. 1:**
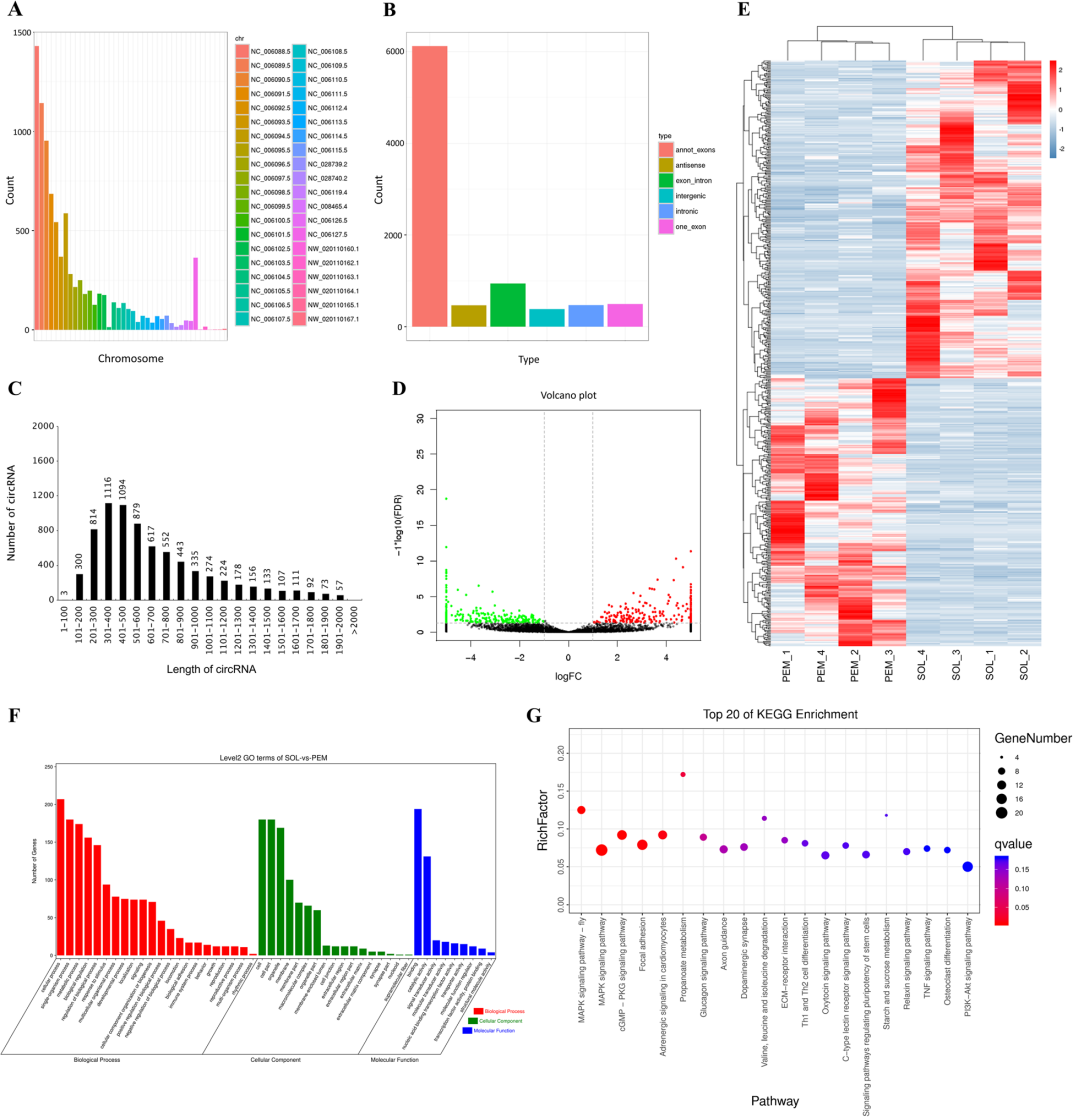
Overview of circular RNA sequencing. (**A**) Chromosome distribution of circRNA transcripts identified in pectoralis major (PEM) and soleus (SOL) of 7-week-old Xinghua chicken. (**B**) Genomic origin of circRNA in PEM and SOL of 7-week-old Xinghua chicken. (**C**) Length distribution of circRNA in PEM and SOL of 7-week-old Xinghua chicken. (**D** and **E**) Volcano plot (**D**) and heatmap (**E**) of differentially expressed circRNA between PEM and SOL in 7-week-old Xinghua chicken. (**F** and **G**) GO functions (**F**) and KEGG pathways (**G**) analysis of the parental genes of differentially expressed circRNAs

In poultry, breast muscle is generally considered to be composed of fast-twitch myofibers, while the leg muscle has a higher proportion of slow-twitch fibers [24, 26]. To systematically identify circRNAs involved in skeletal muscle development, we performed a circRNA-seq to analyze differentially expressed circRNAs between PEM (which is mainly composed of fast-twitch fibers) and SOL (which has higher proportion of slow muscle fibers) in 7-week-old Xinghua chicken. A total of 8882 circRNAs were detected, which were mainly (more than 85%) distributed among chromosomes 1 to 15, and W (Fig. 1A). According to their genomic locus, we found most of them (~ 75%) originate from coding exon (Fig. 1B). The length distribution of those circRNAs is relatively concentrated, with most in the range of 0–2000 nt (Fig. 1C).

In total, 532 circRNAs were identified as being differentially expressed between PEM and SOL in 7-week-old Xinghua chicken (*P* < 0.05; |log_2_FC| > 1) (Table S3). Among the differentially expressed circRNAs, 243 showed upregulation in PEM, while 289 were increased in SOL (Fig. 1D and E; Table S3). Recent studies have found that the biogenesis of circRNA can competes with pre-mRNA splicing, and intron or exon-intron circRNAs can regulate the transcription of their parental gene [27–29]. Next, Gene Ontology (GO) and Kyoto Encyclopedia of Genes and Genomes (KEGG) enrichment analyses were performed for the parental genes of differentially expressed circRNAs. The results showed that these genes were mainly enriched in biological processes such as cellular process, metabolic process, and biological regulation, as well as participated in skeletal muscle development related pathways including MAPK signaling pathway, cGMP-PKG signaling pathway, PI3K-Akt signaling pathway, and so on (Fig. 1F and G).

### *circPTPN4* is a novel circRNA regulated by *FOXA2*

**Fig. 2:**
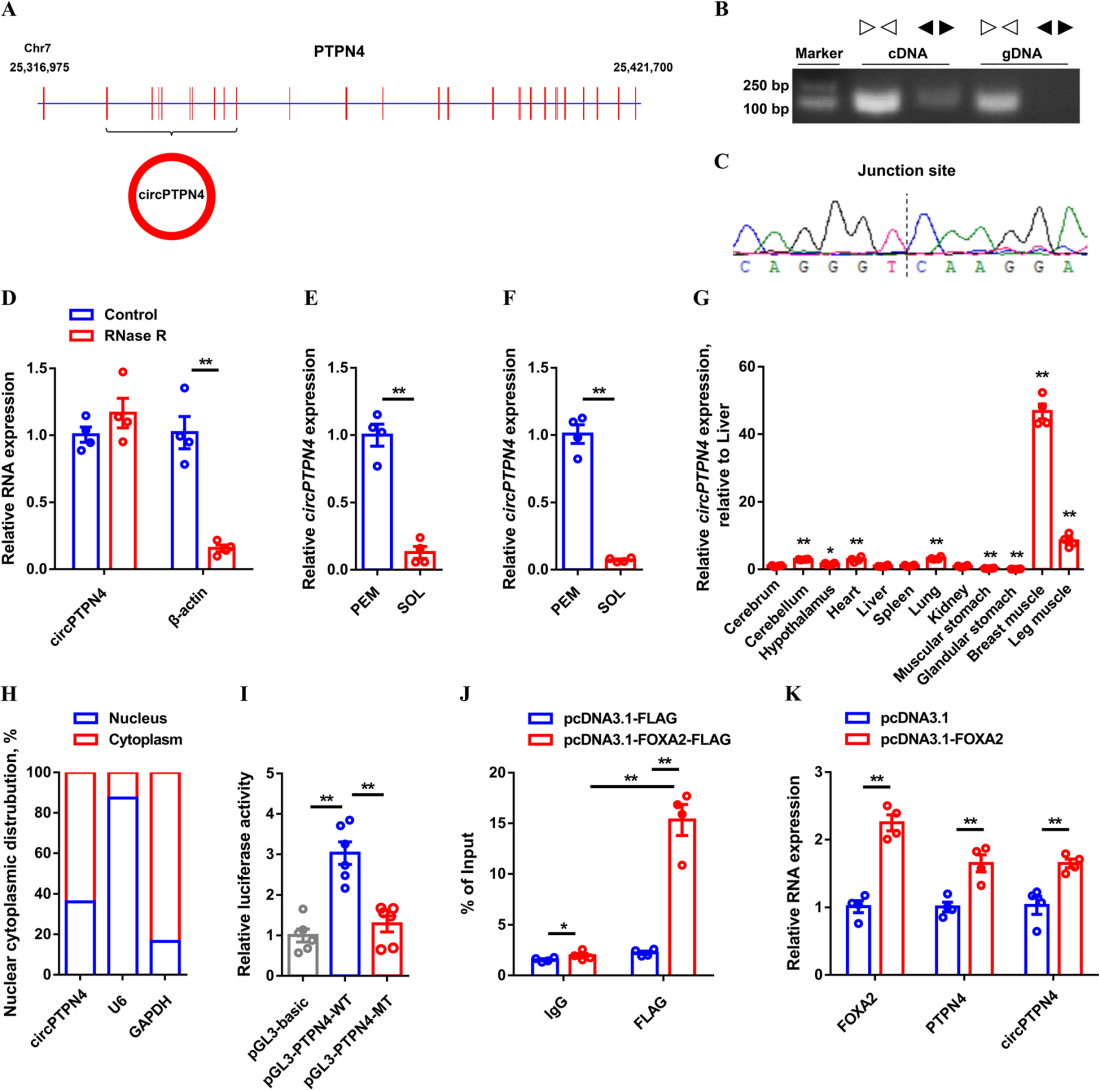
Identification of *circPTPN4*. (**A**) Schematic image of *circPTPN4* derived from *PTPN4*. (**B**) Verification of *circPTPN4* by amplifying with divergent primers. (**C**) Sanger sequencing confirmed the back-splicing junction sequence of *circPTPN4*. (**D**) Relative *circPTPN4* and *β-actin* expression after treatment with RNase R. (**E** and **F**) Relative *circPTPN4* expression in pectoralis major (PEM) and soleus (SOL) of 7-week-old Xinghua chicken detected by RNA-seq (**E**) and qPCR (**F**). (**G**) Tissue expression profiles of *circPTPN4*. The horizontal axis and vertical axis indicate different tissues and their relative expression values, respectively. (**H**) The distribution of *circPTPN4* in the cytoplasm and nuclei of chicken primary myoblast (CPM) was determined by qRT-PCR. *GAPDH* and *U6* serve as cytoplasmic and nuclear localization controls, respectively. (**I**) The transcriptional activity of the *PTPN4* core promoter region. (**J**) Chromatin immunoprecipitation (ChIP) analysis of the binding capacity of FOXA2 to the *PTPN4* core promoter region. (**K**) Relative *FOXA2*, *circPTPN4* and *circPTPN4* expression with *FOXA2* overexpression. Results are presented as mean ± SEM. In panels (**D** to **H**, and **K**), the statistical significance of differences between means was assessed using paired *t*-tests. In panels (**I** and **J**), ANOVA followed by Dunnett’s test was used. (*P < 0.05; **P < 0.01)

To further elucidate the regulation mechanism of circRNA involved in skeletal muscle development, a novel differentially expressed circRNA, *circPTPN4* (novel_circ_002621; which was derived from exon 2–10 of *PTPN4*, highly conserved in *Meleagris gallopavo*, *Numida meleagris* and *Anser cygnoides*) (Fig. 2A and S2; Table S4), was served as a candidate. Firstly, genomic DNA (gDNA) and cDNA were used for the PCR reaction with convergent and divergent primers to confirm the sequence and the junction of *circPTPN4*. A single distinct band with the expected product size was only observed in cDNA samples (Fig. 2B), and the real existence was detected by Sanger sequencing (Fig. 2C). These results suggested that the presence of back-splicing junctions but not genomic rearrangement. Moreover, the RNase R tolerance test showed *circPTPN4* has more resistance than the linear mRNA control (Fig. 2D), which confirmed that *circPTPN4* is a real circRNA. Our circRNA-seq data showed *circPTPN4* was differentially expressed between PEM and SOL in 7-week-old Xinghua chicken (Fig. 2E). Similarly, the consistent result was found by quantitative PCR (qPCR) (Fig. 2F). *circPTPN4* was highly expressed in breast and leg muscle (Fig. 2G), implying that it may play an important role in skeletal muscle development. In addition, cell-fractionation assays demonstrated that *circPTPN4* is mainly present in the cytoplasm of chicken primary myoblast (CPM) (Fig. 2H).

To explore the mechanism through which *circPTPN4* is regulated at the transcriptional level, we further analyzed the core promoter region of *PTPN4* (which is the parental gene of *circPTPN4*), and found a potential binding site for FOXA2 (− 241 to − 228 bp). Dual-luciferase reporter assay confirmed that the mutation of this site leads to a decrease of the transcriptional activity (Fig. 2I and S3A), while the transcriptional activity was increased with *FOXA2* overexpression (Fig. S3B). Moreover, results of a chromatin immunoprecipitation (ChIP) assay confirmed that FOXA2 could physically bind to the core promoter of *PTPN4* (Fig. 2J). Overexpression of *FOXA2* upregulated the expression of *PTPN4* and *circPTPN4* (Fig. 2K). Collectively, these data revealed that *circPTPN4* is positively regulated by the *FOXA2*.

### *circPTPN4* facilitates the proliferation and differentiation of myoblast

**Fig. 3:**
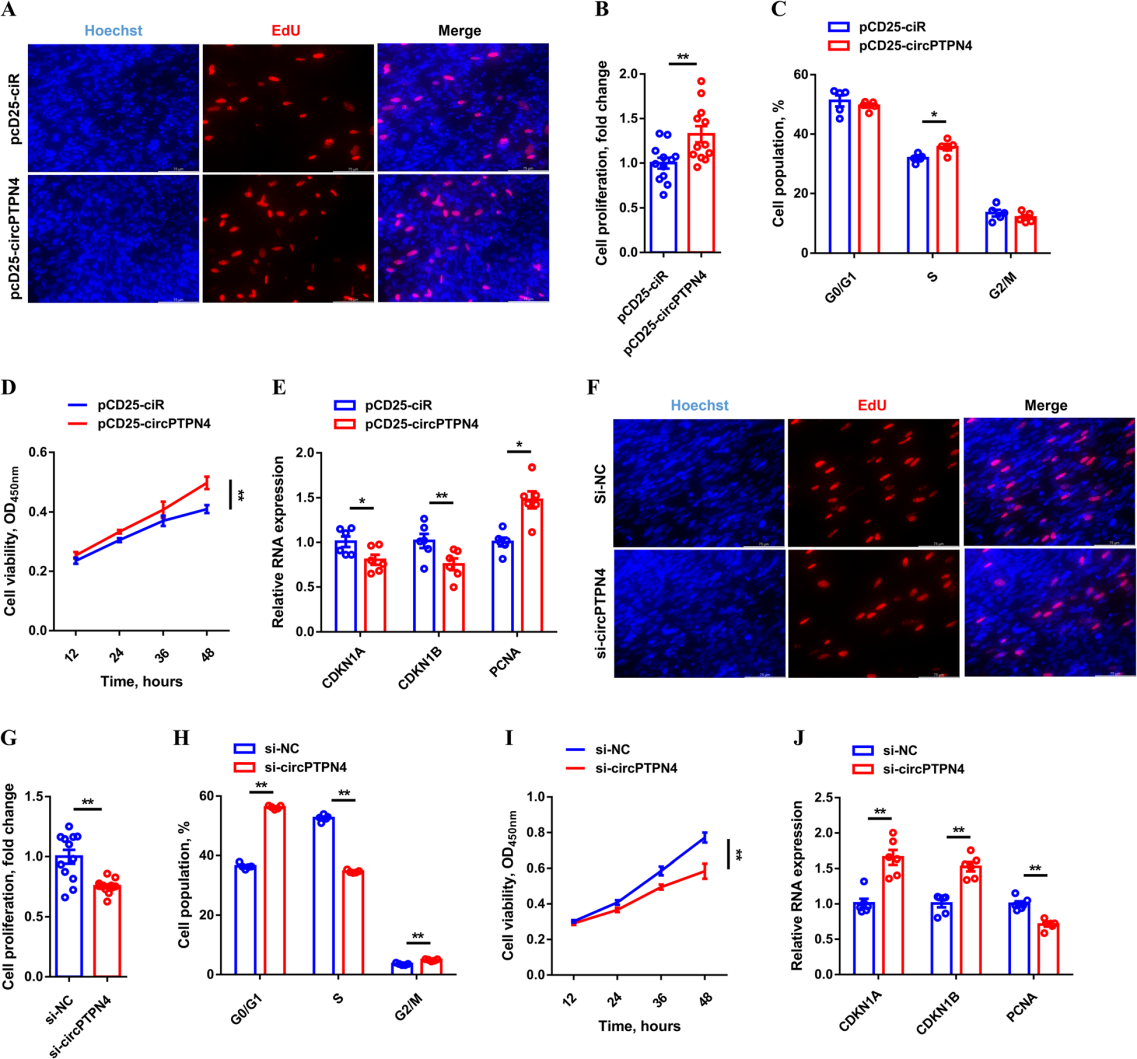
*circPTPN4* promotes myoblast proliferation. (**A** to **E**) EdU proliferation assay (**A**), the proliferation rate of myoblast (**B**), cell cycle analysis (**C**), CCK-8 assay (**D**), and relative mRNA levels of several cell cycle genes (**E**) with *circPTPN4* overexpression in CPMs. (**F** to **J**) EdU proliferation assay (**F**), proliferation rate of myoblast (**G**), cell cycle analysis (**H**), CCK-8 assay (**I**), and relative mRNA levels of several cell cycle genes (**J**) after *circPTPN4* interference in CPMs. Results are shown as mean ± SEM. In panels (**B** to **E**, and **G** to **J**), the statistical significance of differences between means was assessed using independent sample *t*-test. (*P < 0.05; **P < 0.01)

In order to assess the function of *circPTPN4* in myogenesis, the overexpression vector of *circPTPN4* was constructed and transfected into CPM (Fig. S4A). The 5-ethynyl-2′-deoxyuridine (EdU) staining demonstrated that *circPTPN4* overexpression significantly increased EdU incorporation and promoted myoblast proliferation (Fig. 3A and B). Flow cytometric analysis and cell counting kit-8 (CCK-8) assay also showed that overexpression of *circPTPN4* significantly increased the number of S phase cells (Fig. 3C), and improved myoblast viability (Fig. 3D). Furthermore, *circPTPN4* overexpression repressed the expression level of cell cycle-inhibiting genes, including *CDKN1A* and *CDKN1B*, while increasing the expression level of cell cycle-promoting genes like *PCNA* (Fig. 3E). Conversely, the opposite result was observed by *circPTPN4* interference (Fig. 3F to J, and S4B), indicating that *circPTPN4* can facilitate myoblast proliferation.

**Fig. 4:**
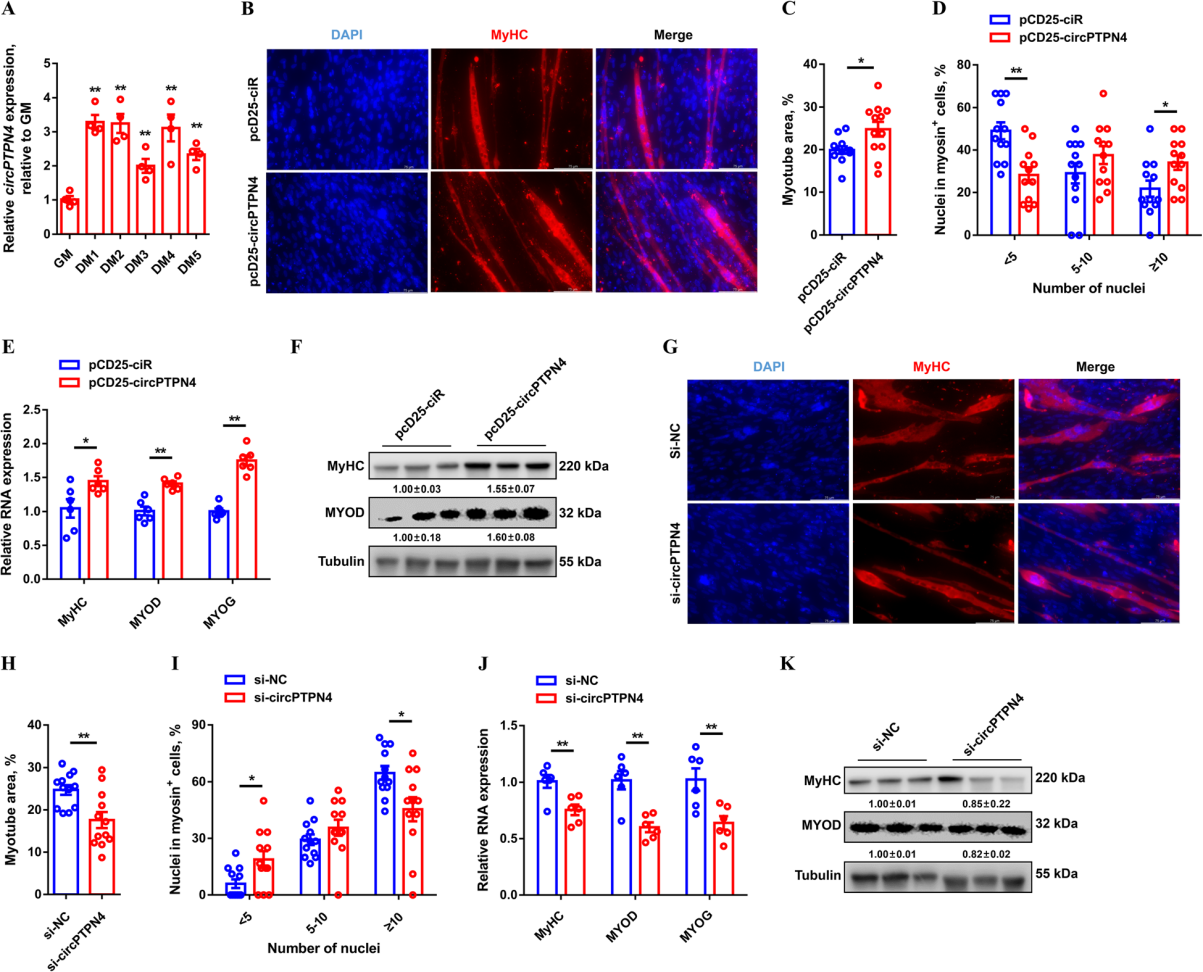
*circPTPN4* induces myogenetic differentiation. (**A**) Relative *circPTPN4* expression during CPM differentiation. (**B** to **F**) MyHC immunostaining (**B**), myotube area (**C**), myoblast fusion index (**D**) and relative mRNA (**E**) and protein (**F**) expression levels of myoblast differentiation marker genes after overexpression of *circPTPN4*. (**G** to **K**) MyHC immunostaining (**G**), myotube area (**H**), myoblast fusion index (**I**) and relative mRNA (**J**) and protein (**K**) expression levels of myoblast differentiation marker genes with *circPTPN4* inhibition. In panels (**F** and **K**), the numbers shown below the bands were folds of band intensities relative to control. Band intensities were quantified by ImageJ and normalized to β-Tubulin. Data are expressed as a fold-change relative to the control. Results are shown as mean ± SEM. In panels (**A**, **C** to **E**, and **H** to **J**), the statistical significance of differences between means was assessed using independent sample *t*-test. (*P < 0.05; **P < 0.01)

*circPTPN4* expression was upregulated with myogenic differentiation (Fig. 4A), which suggested that *circPTPN4* may be involved in the process of myoblast differentiation. To further investigate the potential function of *circPTPN4*, immunofluorescence staining was performed. Immunofluorescence staining showed that overexpression of *circPTPN4* increased the total areas of myotubes and induced myotube formation (Fig. 4B to D). In addition, the expressions level of myoblast differentiation marker genes, including *MyHC*, *MYOD*, and *MYOG* were significantly upregulated with *circPTPN4* overexpression (Fig. 4E and F). On the contrary, *circPTPN4* interference decreased the total areas of myotubes and inhibited myoblast fusion, as well as downregulated the expression of myoblast differentiation marker genes (Fig. 4G to K).

### *circPTPN4* suppresses mitochondria biogenesis and activates fast-twitch muscle phenotype

**Fig. 5:**
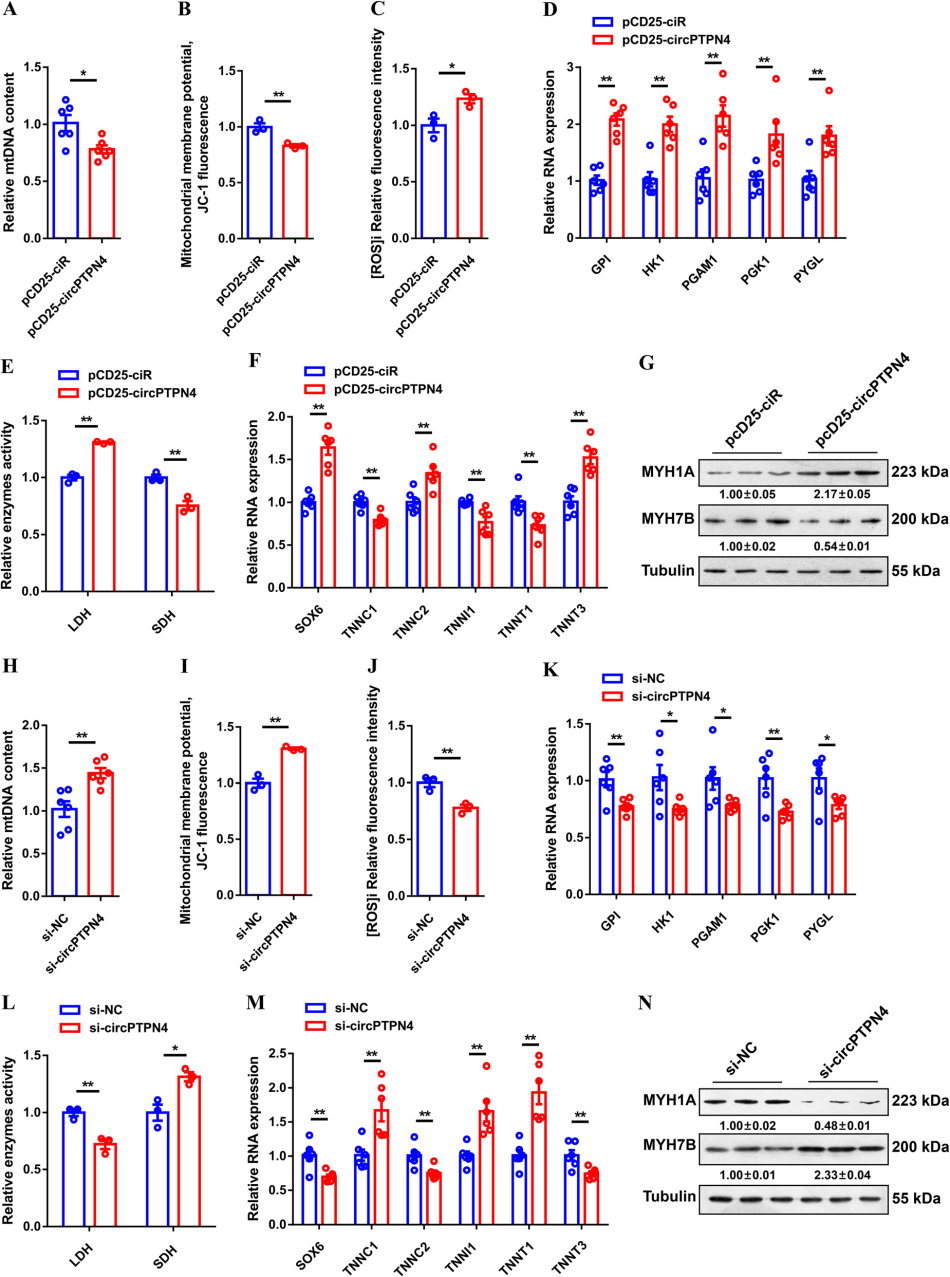
*circPTPN4* represses mitochondria biogenesis and drives the transformation of slow-twitch to fast-twitch myofiber. (**A** to **G**) Relative mitochondrial DNA (mtDNA) content (**A**), mitochondrial membrane potential (**B**), reactive oxygen species (ROS) production (**C**), relative mRNA expression levels of glycogenolytic and glycolytic genes (**D**), relative enzymes activity of lactic dehydrogenase (LDH) and succinate dehydrogenase (SDH) (**E**), relative mRNA expression levels of several fast−/slow-twitch myofiber genes (**F**), and relative protein expression of MYH1A and MYH7B (**G**) in *circPTPN4* overexpression CPMs. (**H** to **N**) Relative mtDNA content (**H**), mitochondrial membrane potential (**I**), ROS production (**J**), relative mRNA expression levels of glycogenolytic and glycolytic genes (**K**), relative enzymes activity of LDH and SDH (**L**), relative mRNA expression levels of several fast−/slow-twitch myofiber genes (**M**), and relative protein expression of MYH1A and MYH7B (**N**) in CPMs with *circPTPN4* interference. In panels (**G** and **N**), the numbers shown below the bands were folds of band intensities relative to control. Band intensities were quantified by ImageJ and normalized to β-Tubulin. Data are expressed as a fold-change relative to the control. In all panels, results are shown as mean ± SEM, the statistical significance of differences between means was assessed using independent sample *t*-test. (*P < 0.05; **P < 0.01)

Skeletal muscle is a major player in regulating energy homeostasis [30, 31]. As the main organelle of energy metabolism, mitochondria are closely related to the development of skeletal muscle [32, 33]. Next, we evaluated mitochondrial content and function after overexpression and inhibition of *circPTPN4*. *circPTPN4* overexpression decreased mitochondrial DNA (mtDNA) content and was accompanied by a decline of mitochondrial membrane potential (Fig. 5A and B). Meanwhile, reactive oxygen species (ROS) production was significantly increased after *circPTPN4* overexpression (Fig. 5C). Inversely, *circPTPN4* inhibition increased mitochondrial content and enhanced mitochondrial function (Fig. 5H to J), illustrating that *circPTPN4* suppresses mitochondria biogenesis.

Skeletal muscle is comprised of heterogeneous myofibers that differ in their physiological and metabolic parameters [34]. Compared with slow-twitch (type I; oxidative) myofibers, fast-twitch (type II; glycolytic) myofibers have fewer mitochondria and higher activity of glycolytic metabolic enzymes [35, 36]. Given that *circPTPN4* is highly expressed in fast-twitch myofiber and repressed mitochondria biogenesis, we speculated that *circPTPN4* may function in the activization of fast-twitch muscle phenotype. As expected, overexpression of *circPTPN4* upregulated the expression of glycogenolytic and glycolytic genes (Fig. 5D). The activity of lactate dehydrogenase (LDH) was enhanced, while the activity of succinate dehydrogenase (SDH) was suppressed with *circPTPN4* overexpression (Fig. 5E). *circPTPN4* overexpression upregulated expressions of multiple fast-twitch myofiber genes like *SOX6*, *TNNC2* and *TNNT3*, while suppressed slow-twitch myofiber genes such as *TNNC1*, *TNNI1* and *TNNT1* (Fig. 5F). More importantly, western blot results showed that overexpression of *circPTPN4* promoted MYH1A/fast-twitch protein level and suppressed the expression level of MYH7B/slow-twitch protein (Fig. 5G). On the contrary, the glycolytic capacity of skeletal muscle was suppressed and the slow-twitch muscle phenotype was induced with *circPTPN4* interference (Fig. 5K to N).

### *circPTPN4* interacts with *miR-449-3p* to upregulate *NAMPT* expression, thus inactivating AMPK signaling

**Fig. 6:**
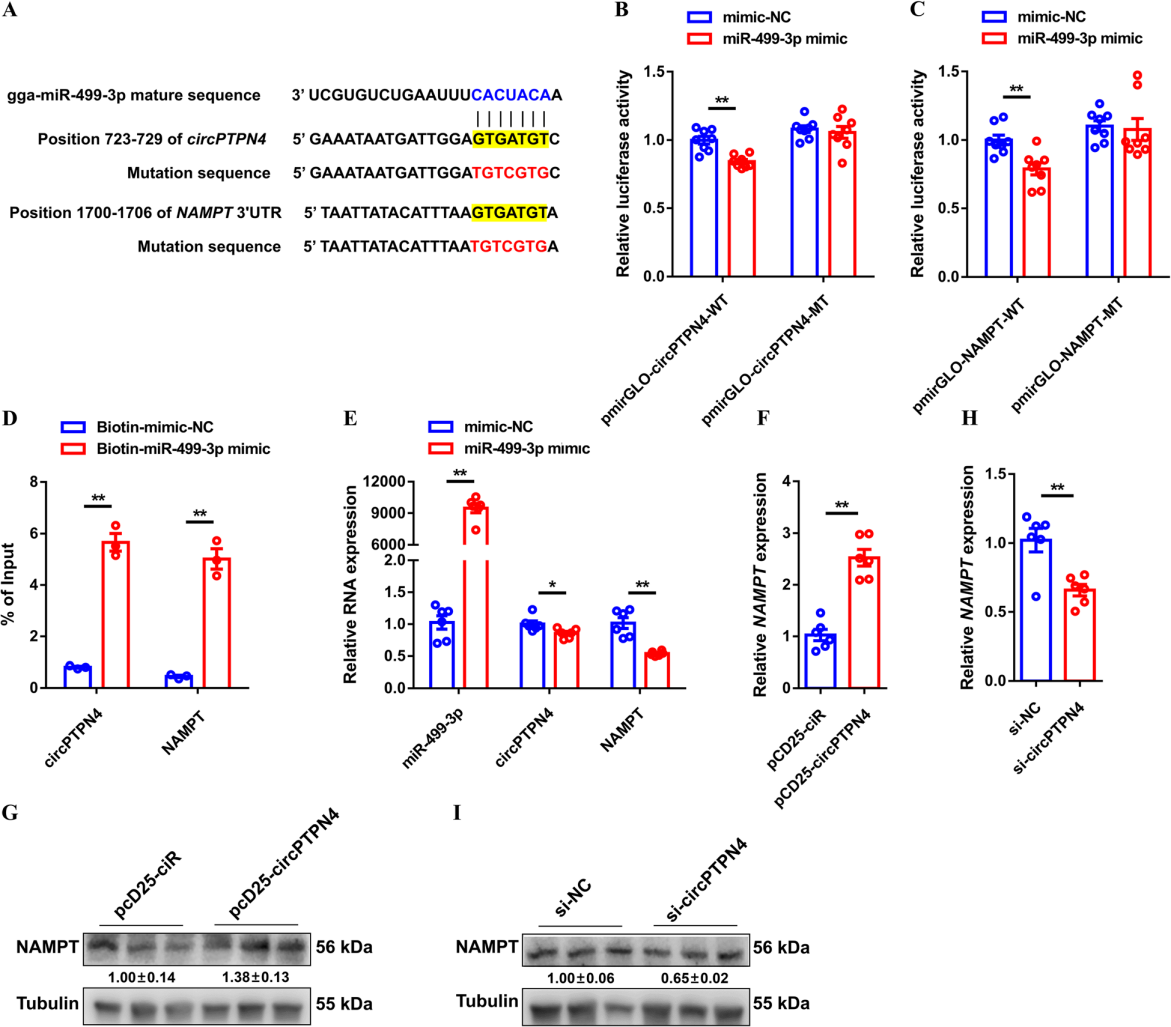
*circPTPN4* functions as a competing endogenous RNA (ceRNA) to regulate *NAMPT* expression by sponging *miR-499-3p*. (**A**) The potential binding site of *miR-499-3p* in *circPTPN4* transcript and *NAMPT* 3′ untranslated region (UTR). The mutant sequence in *miR-499-3p* binding site is highlighted in red. (**B** and **C**) Dual-luciferase reporter assay was conducted by co-transfecting the wild type or mutant: (**B**) *circPTPN4* and (**C**) *NAMPT* 3′ UTR with a *miR-499-3p* mimic or mimic-negative control (NC). (**D**) The interaction of *miR-499-3p* with *circPTPN4* and *NAMPT* was determined by biotin-coupled miRNA pull down. (**E**) Relative *miR-499-3p*, *circPTPN4*, and *NAMPT* expression after overexpression of *miR-499-3p*. (**F** and **G**) Relative mRNA (**F**) and protein (**G**) expression levels of *NAMPT* with *circPTPN4* overexpression. (**H** and **I**) Relative mRNA (**H**) and protein (**I**) expression levels of *NAMPT* after *circPTPN4* interference. In panels (**G** and **I**), the numbers shown below the bands were folds of band intensities relative to control. Band intensities were quantified by ImageJ and normalized to β-Tubulin. Data are expressed as a fold-change relative to the control. Results are presented as mean ± SEM. In panels (**B** to **H**), the statistical significance of differences between means was assessed using independent sample *t*-test. (*P < 0.05; **P < 0.01)

In 2011, competitive endogenous RNAs (ceRNAs) were first reported as endogenous sponges that can affect the distribution of miRNAs on their targets, thereby imposing another novel layer of posttranscriptional regulation [37]. Given that *circPTPN4* is mainly present in the cytoplasm, we hypothesized that *circPTPN4* may function as a ceRNA to exert its biological function. The target miRNAs and genes of *circPTPN4* were predicted on an RNAhybrid software. Interestingly, *miR-499-3p* was found to contain potential binding sites for both *circPTPN4* and *NAMPT* (Fig. 6A), suggesting that it may mediate the regulation of *NAMPT* expression by *circPTPN4*. Compared with PEM, the expression of *miR-499-3p* is higher in SOL (Fig. S5A). In contrast, *NAMPT* is highly expressed in PEM (Fig. S5B), which is consistent with *circPTPN4*, further hinting that the interaction of *circPTPN4* with *miR-499-3p* and *NAMPT*. Dual-luciferase reporter assays were carried out to confirm whether *miR-499-3p* directly interact with *circPTPN4* and *NAMPT*. The results showed that *miR-499-3p* bind with both *circPTPN4* and the 3′ UTR of *NAMPT* (Fig. 6B to C). Furthermore, the interaction of *miR-499-3p* with *circPTPN4* and *NAMPT* was also verified by a biotin-coupled miRNA pull down assay (Fig. 6D). Overexpression of *miR-499-3p* repressed the expression of *circPTPN4* and *NAMPT* (Fig. 6E). More importantly, *circPTPN4* overexpression upregulated the mRNA and protein levels of *NAMPT*, whereas the expression of *NAMPT* was suppressed with *circPTPN4* interference (Fig. 6F-I), explaining the targeted regulation of *circPTPN4* on *NAMPT*.

**Fig. 7:**
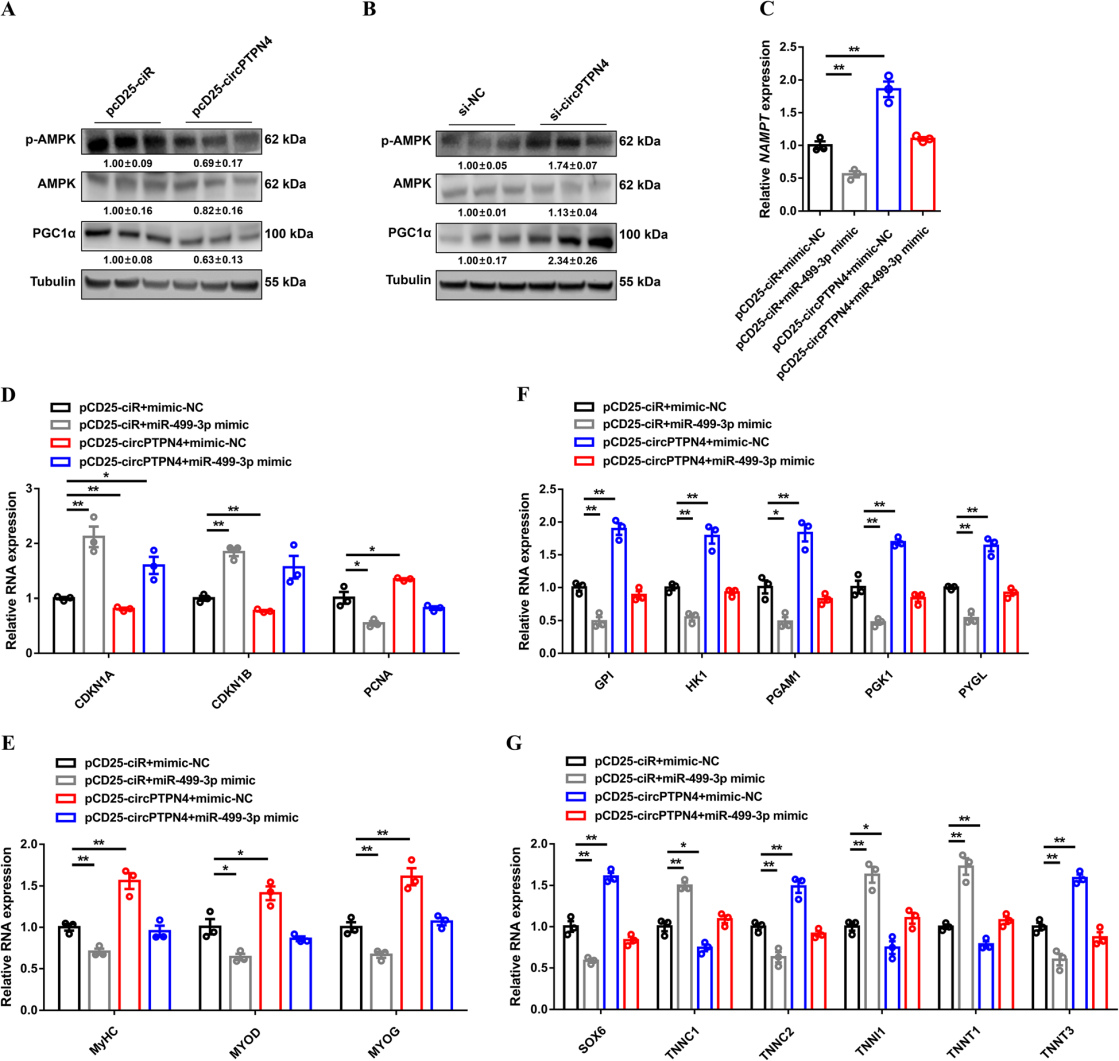
The *miR-499-3p*/*NAMPT*/*AMPK* axis is required for the function of *circPTPN4*. (**A** and **B**) Protein expression levels of AMPK signaling with *circPTPN4* overexpression (**A**) or interference (**B**). The numbers shown below the bands were folds of band intensities relative to control. Band intensities were quantified by ImageJ and normalized to β-Tubulin. Data are expressed as a fold-change relative to the control. (**C** to **G**) Relative mRNA expression of *NAMPT* (**C**), relative mRNA levels of several cell cycle genes (**D**), relative mRNA expression levels of myoblast differentiation marker genes (**E**), relative mRNA expression levels of glycogenolytic and glycolytic genes (**F**), and relative mRNA expression levels of several fast−/slow-twitch myofiber genes (**G**) induced by the listed nucleic acids in CPMs. Results are shown as mean ± SEM. In panels (**C** to **G**), the statistical significance of differences between means was assessed using ANOVA followed by Dunnett’s test. (*P < 0.05; **P < 0.01)

Previous studies have shown that *NAMPT* is widely involved in a series of biological processes by inactivating AMPK signaling [38–40]. We further assessed the AMPK signaling and found that *circPTPN4* overexpression inhibited the phosphorylation of AMPK and downregulated the expression of PGC1α (Fig. 7A). Conversely, this pathway was activated with the interference of *circPTPN4* (Fig. 7B), suggesting that *circPTPN4* may participate in AMPK signaling by regulating *NAMPT*. Overexpression of *circPTPN4* alleviated the inhibitory effect of *miR-499-3p* on *NAMPT* expression (Fig. 7C). In addition, the regulatory effects of *circPTPN4* were weakened after *miR-499-3p* overexpression (Fig. 7D to G), indicating that the *miR-499-3p*/*NAMPT*/*AMPK* axis is required for the function of *circPTPN4*.

## Discussion

Due to the low quantity and expressive abundance, circRNAs were once considered to be an abnormal splicing product of RNA or unique structure of pathogens, with less attention [41, 42]. However, recent studies have found that circRNA is universally present in archaea, suggesting that it may have important biological functions [43]. With the development of genome research, more and more circRNAs are found in various cells and tissues, which are widely present in eukaryotes [44–47]. In this study, a total of 532 circRNAs were identified as being differentially expressed between PEM and SOL in 7-week-old Xinghua chicken. Among them, a novel differentially expressed circRNA, *circPTPN4*, was served as a candidate. *circPTPN4* is highly expressed in fast-twitch myofiber, and its expression upregulates with myoblast differentiation, suggesting that it may play a significant role in skeletal muscle development.

Myogenesis is a process including myoblast proliferation, differentiation and myotube formation and is controlled by a series of myogenic regulatory factors. These factors can regulate myoblasts to withdraw from the cell cycle, express muscle-specific genes, and prevent the expression of other cell- or tissue-specific genes. Recently, it is worth noting that circRNAs have also been demonstrated to function in myogenesis [48–51]. Here, we found that *circPTPN4* promotes myoblast proliferation and induces myogenic differentiation.

Skeletal muscle is composed of different types of myofibers. Under certain conditions, different types of myofibers can be transformed. Previous studies have found that a total of 305 circRNAs were differentially expressed between the oxidative muscle sartorius compared and the glycolytic muscle pectoralis major in Chinese Qingyuan partridge chickens. Among them, *novel_circ_004282* and *novel_circ_002121* were speculated to play important roles in regulating oxidative myofibers by *PPP3CA* and *NFATC1* expression [52]. As a transcriptional coactivator, PGC1α is a downstream effector of AMPK signaling, has been found to regulate mitochondria biogenesis and the transformation of myofiber type [53–55]. In the current study, we found that *circPTPN4* decreases mtDNA content and suppresses mitochondria functions. Moreover, *circPTPN4* improves the glycolytic capacity of myoblast to activate fast-twitch muscle phenotype, demonstrating that *circPTPN4* is involved in the transformation of myofiber type by inactivating AMPK signaling.

Recently, a new pattern of gene expression has been come up with regarding the interaction of RNA transcripts, called ceRNA [37]. There is a great deal of researches indicated that circRNAs can function as ceRNAs to protect mRNAs by acting as molecular sponges for miRNAs, thereby modulating the de-repression of miRNA targets and imposing an additional level of post-transcriptional regulation [46, 49, 50]. In this study, using in silico analysis, we found *miR-499-3p* contains binding sites for *circPTPN4* and *NAMPT*. The interaction of *miR-499-3p* with *circPTPN4* and *NAMPT* was further validated by dual-luciferase reporter assay and biotin-coupled miRNA pull down assay. *circPTPN4* regulates *NAMPT* expression to function in AMPK signaling. In addition, our rescue experiment showed that the biological functions of *circPTPN4* were weakened with *miR-499-3p* overexpression, explaining that the *miR-499-3p*/*NAMPT* axis is required for the function of *circPTPN4*.

## Conclusions

**Fig. 8:**
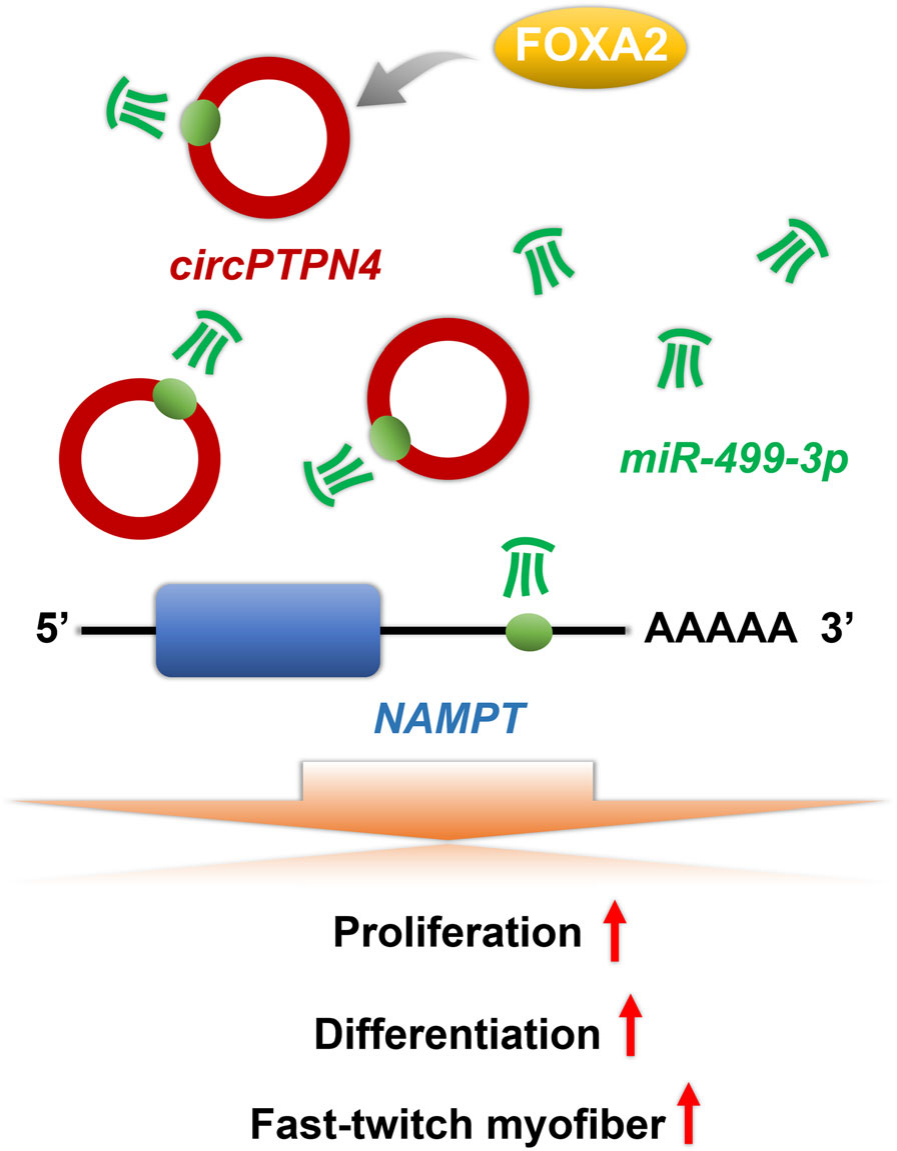
Model of *circPTPN4* functions as a ceRNA to regulate *NAMPT* expression by sponging *miR-499-3p*, thus promoting the proliferation and differentiation of myoblast, as well as activating fast-twitch muscle phenotype

In conclusion, we demonstrated that *circPTPN4* is a novel circRNA, which is highly expressed in fast-twitch myofiber and is positively regulated by transcription factor FOXA2. Mechanistically, *circPTPN4* can function as a ceRNA to regulate *NAMPT* expression by sponging *miR-499-3p*, thus promoting the proliferation and differentiation of myoblast, as well as activating fast-twitch muscle phenotype (Fig. 8). Our findings provide a solid foundation for the understanding of the mechanisms and regulatory networks of myogenesis, and will contribute to the development of further research.

## Supplementary Information

**Additional file 1.**

## Data Availability

The data were exhibited in the main manuscript and supplemental materials.

## Abbreviations

CCK-8: Cell counting kit-8
ceRNA: Competing endogenous RNA
ChIP: Chromatin immunoprecipitation
circRNA: Circular RNA
CPMs: Chicken primary myoblasts
EdU: 5-Ethynyl-2′-deoxyuridine
gDNA: Genomic DNA
GO: Gene Ontology
IF: Immunofluorescence
KEGG: Kyoto Encyclopedia of Genes and Genomes
LDH: Lactic dehydrogenase
miRNA: MicroRNA
mtDNA: Mitochondrial DNA
*NAMPT*: Nicotinamide phosphoribosyltransferase
NC: Negative control
ncRNA: Noncoding RNA
PEM: Pectoralis major
ROS: Reactive oxygen species
SDH: Succinate dehydrogenase
siRNA: Small interfering RNA
SOL: Soleus
UTR: Untranslated region

## References

[1] Lee SH, Joo ST, Ryu YC (2010). Skeletal muscle fiber type and myofibrillar proteins in relation to meat quality. Meat Sci.

[2] Joo ST, Kim GD, Hwang YH, Ryu YC (2013). Control of fresh meat quality through manipulation of muscle fiber characteristics. Meat Sci.

[3] Wilkins MR, Sanchez JC, Gooley AA, Appel RD, Humphery-Smith I, Hochstrasser DF, Williams KL (1996). Progress with proteome projects: why all proteins expressed by a genome should be identified and how to do it. Biotechnol Genet Eng Rev.

[4] Djebali S, Davis CA, Merkel A, Dobin A, Lassmann T, Mortazavi A, Tanzer A, Lagarde J, Lin W, Schlesinger F, Xue C, Marinov GK, Khatun J, Williams BA, Zaleski C, Rozowsky J, Röder M, Kokocinski F, Abdelhamid RF, Alioto T, Antoshechkin I, Baer MT, Bar NS, Batut P, Bell K, Bell I, Chakrabortty S, Chen X, Chrast J, Curado J, Derrien T, Drenkow J, Dumais E, Dumais J, Duttagupta R, Falconnet E, Fastuca M, Fejes-Toth K, Ferreira P, Foissac S, Fullwood MJ, Gao H, Gonzalez D, Gordon A, Gunawardena H, Howald C, Jha S, Johnson R, Kapranov P, King B, Kingswood C, Luo OJ, Park E, Persaud K, Preall JB, Ribeca P, Risk B, Robyr D, Sammeth M, Schaffer L, See LH, Shahab A, Skancke J, Suzuki AM, Takahashi H, Tilgner H, Trout D, Walters N, Wang H, Wrobel J, Yu Y, Ruan X, Hayashizaki Y, Harrow J, Gerstein M, Hubbard T, Reymond A, Antonarakis SE, Hannon G, Giddings MC, Ruan Y, Wold B, Carninci P, Guigó R, Gingeras TR (2012). Landscape of transcription in human cells. Nature..

[5] Xu M, Chen X, Chen D, Yu B, Li M, He J, Huang Z (2020). Regulation of skeletal myogenesis by microRNAs. J Cell Physiol.

[6] Chen R, Lei S, Jiang T, Zeng J, Zhou S, She Y (2020). Roles of lncRNAs and circRNAs in regulating skeletal muscle development. Acta Physiol (Oxford).

[7] Memczak S, Jens M, Elefsinioti A, Torti F, Krueger J, Rybak A, Maier L, Mackowiak SD, Gregersen LH, Munschauer M, Loewer A, Ziebold U, Landthaler M, Kocks C, le Noble F, Rajewsky N (2013). Circular RNAs are a large class of animal RNAs with regulatory potency. Nature..

[8] Rybak-Wolf A, Stottmeister C, Glazar P, Jens M, Pino N, Giusti S (2015). Circular RNAs in the mammalian brain are highly abundant, conserved, and dynamically expressed. Mol Cell.

[9] Chen LL (2016). The biogenesis and emerging roles of circular RNAs. Nat Rev Mol Cell Biol.

[10] Dong R, Zhang XO, Zhang Y, Ma XK, Chen LL, Yang L (2016). CircRNA-derived pseudogenes. Cell Res.

[11] Bach DH, Lee SK, Sood AK (2019). Circular RNAs in cancer. Mol Ther Nucleic Acids.

[12] Wu J, Qi X, Liu L, Hu X, Liu J, Yang J, Yang J, Lu L, Zhang Z, Ma S, Li H, Yun X, Sun T, Wang Y, Wang Z, Liu Z, Zhao W (2019). Emerging epigenetic regulation of circular RNAs in human cancer. Mol Ther Nucleic Acids.

[13] Bartel DP (2004). MicroRNAs: genomics, biogenesis, mechanism, and function. Cell..

[14] Wahid F, Shehzad A, Khan T, Kim YY (2010). MicroRNAs: synthesis, mechanism, function, and recent clinical trials. Biochim Biophys Acta.

[15] Liu X, Zhang Y, Liang H, Zhang Y, Xu Y (2020). MicroRNA-499-3p inhibits proliferation and promotes apoptosis of retinal cells in diabetic retinopathy through activation of the TLR4 signaling pathway by targeting IFNA2. Gene..

[16] Jiang A, Yin D, Zhang L, Li B, Li R, Zhang X, Zhang Z, Liu H, Kim K, Wu W (2021). Parsing the microRNA genetics basis regulating skeletal muscle fiber types and meat quality traits in pigs. Anim Genet.

[17] Samal B, Sun Y, Stearns G, Xie C, Suggs S, McNiece I (1994). Cloning and characterization of the cDNA encoding a novel human pre-B-cell colony-enhancing factor. Mol Cell Biol.

[18] Imai S (2009). Nicotinamide phosphoribosyltransferase (Nampt): a link between NAD biology, metabolism, and diseases. Curr Pharm Des.

[19] Garten A, Schuster S, Penke M, Gorski T, de Giorgis T, Kiess W (2015). Physiological and pathophysiological roles of NAMPT and NAD metabolism. Nat Rev Endocrinol.

[20] Cai B, Ma M, Chen B, Li Z, Abdalla BA, Nie Q, Zhang X (2018). MiR-16-5p targets SESN1 to regulate the p53 signaling pathway, affecting myoblast proliferation and apoptosis, and is involved in myoblast differentiation. Cell Death Dis.

[21] Jeck WR, Sorrentino JA, Wang K, Slevin MK, Burd CE, Liu J, Marzluff WF, Sharpless NE (2013). Circular RNAs are abundant, conserved, and associated with ALU repeats. RNA..

[22] Cai B, Li Z, Ma M, Zhang J, Kong S, Abdalla BA, Xu H, Jebessa E, Zhang X, Lawal RA, Nie Q (2021). Long noncoding RNA SMUL suppresses SMURF2 production-mediated muscle atrophy via nonsense-mediated mRNA decay. Mol Ther Nucleic Acids.

[23] Cai B, Li Z, Ma M, Wang Z, Han P, Abdalla BA, Nie Q, Zhang X (2017). LncRNA-Six1 encodes a micropeptide to activate six1 in cis and is involved in cell proliferation and muscle growth. Front Physiol.

[24] Ma M, Cai B, Jiang L, Abdalla BA, Li Z, Nie Q, Zhang X (2018). LncRNA-Six1 is a target of miR-1611 that functions as a ceRNA to regulate six1 protein expression and fiber type switching in chicken myogenesis. Cells..

[25] Orom UA, Lund AH (2007). Isolation of microRNA targets using biotinylated synthetic microRNAs. Methods..

[26] Li L, Liu HH, Xu F, Si JM, Jia J, Wang JW (2010). MyoD expression profile and developmental differences of leg and breast muscle in Peking duck (Anas platyrhynchos Domestica) during embryonic to neonatal stages. Micron..

[27] Zhang Y, Zhang XO, Chen T, Xiang JF, Yin QF, Xing YH, Zhu S, Yang L, Chen LL (2013). Circular intronic long noncoding RNAs. Mol Cell.

[28] Ashwal-Fluss R, Meyer M, Pamudurti NR, Ivanov A, Bartok O, Hanan M, Evantal N, Memczak S, Rajewsky N, Kadener S (2014). CircRNA biogenesis competes with pre-mRNA splicing. Mol Cell.

[29] Li Z, Huang C, Bao C, Chen L, Lin M, Wang X, Zhong G, Yu B, Hu W, Dai L, Zhu P, Chang Z, Wu Q, Zhao Y, Jia Y, Xu P, Liu H, Shan G (2015). Exon-intron circular RNAs regulate transcription in the nucleus. Nat Struct Mol Biol.

[30] Zurlo F, Larson K, Bogardus C, Ravussin E (1990). Skeletal muscle metabolism is a major determinant of resting energy expenditure. J Clin Invest.

[31] Ibrahim A, Neinast M, Arany ZP (2017). Myobolites: muscle-derived metabolites with paracrine and systemic effects. Curr Opin Pharmacol.

[32] Boengler K, Kosiol M, Mayr M, Schulz R, Rohrbach S (2017). Mitochondria and ageing: role in heart, skeletal muscle and adipose tissue. J Cachexia Sarcopenia Muscle.

[33] Boncompagni S, Pozzer D, Viscomi C, Ferreiro A, Zito E (2020). Physical and functional cross talk between Endo-sarcoplasmic reticulum and mitochondria in skeletal muscle. Antioxid Redox Signal.

[34] Bassel-Duby R, Olson EN (2006). Signaling pathways in skeletal muscle remodeling. Annu Rev Biochem.

[35] Schiaffino S, Reggiani C (2011). Fiber types in mammalian skeletal muscles. Physiol Rev.

[36] Koutakis P, Weiss DJ, Miserlis D, Shostrom VK, Papoutsi E, Ha DM, Carpenter LA, McComb RD, Casale GP, Pipinos II (2014). Oxidative damage in the gastrocnemius of patients with peripheral artery disease is myofiber type selective. Redox Biol.

[37] Salmena L, Poliseno L, Tay Y, Kats L, Pandolfi PP (2011). A ceRNA hypothesis: the Rosetta stone of a hidden RNA language?. Cell..

[38] Schuster S, Penke M, Gorski T, Gebhardt R, Weiss TS, Kiess W, Garten A (2015). FK866-induced NAMPT inhibition activates AMPK and downregulates mTOR signaling in hepatocarcinoma cells. Biochem Biophys Res Commun.

[39] Tateishi K, Iafrate AJ, Ho Q, Curry WT, Batchelor TT, Flaherty KT, Onozato ML, Lelic N, Sundaram S, Cahill DP, Chi AS, Wakimoto H (2016). Myc-driven glycolysis is a therapeutic target in glioblastoma. Clin Cancer Res.

[40] Nacarelli T, Lau L, Fukumoto T, Zundell J, Fatkhutdinov N, Wu S, Aird KM, Iwasaki O, Kossenkov AV, Schultz D, Noma KI, Baur JA, Schug Z, Tang HY, Speicher DW, David G, Zhang R (2019). NAD(+) metabolism governs the proinflammatory senescence-associated secretome. Nat Cell Biol.

[41] Kos A, Dijkema R, Arnberg AC, van der Meide PH, Schellekens H (1986). The hepatitis delta (delta) virus possesses a circular RNA. Nature..

[42] Cocquerelle C, Mascrez B, Hetuin D, Bailleul B (1993). Mis-splicing yields circular RNA molecules. FASEB J.

[43] Danan M, Schwartz S, Edelheit S, Sorek R (2012). Transcriptome-wide discovery of circular RNAs in Archaea. Nucleic Acids Res.

[44] Salzman J, Gawad C, Wang PL, Lacayo N, Brown PO (2012). Circular RNAs are the predominant transcript isoform from hundreds of human genes in diverse cell types. PLoS One.

[45] Werfel S, Nothjunge S, Schwarzmayr T, Strom TM, Meitinger T, Engelhardt S (2016). Characterization of circular RNAs in human, mouse and rat hearts. J Mol Cell Cardiol.

[46] Zhang L, Liu X, Che S, Cui J, Ma X, An X, Cao B, Song Y (2019). Endometrial epithelial cell apoptosis is inhibited by a ciR8073-miR181a-Neurotensis pathway during embryo implantation. Mol Ther Nucleic Acids.

[47] Jiang R, Li H, Yang J, Shen X, Song C, Yang Z, Wang X, Huang Y, Lan X, Lei C, Chen H (2020). CircRNA profiling reveals an abundant circFUT10 that promotes adipocyte proliferation and inhibits adipocyte differentiation via sponging let-7. Mol Ther Nucleic Acids.

[48] Chen B, Yu J, Guo L, Byers MS, Wang Z, Chen X, Xu H, Nie Q (2019). Circular RNA circHIPK3 promotes the proliferation and differentiation of chicken myoblast cells by sponging miR-30a-3p. Cells..

[49] Peng S, Song C, Li H, Cao X, Ma Y, Wang X, Huang Y, Lan X, Lei C, Chaogetu B, Chen H (2019). Circular RNA SNX29 sponges miR-744 to regulate proliferation and differentiation of myoblasts by activating the Wnt5a/ca (2+) signaling pathway. Mol Ther Nucleic Acids.

[50] Shen X, Zhang X, Ru W, Huang Y, Lan X, Lei C, Chen H (2020). CircINSR promotes proliferation and reduces apoptosis of embryonic myoblasts by sponging miR-34a. Mol Ther Nucleic Acids.

[51] Yue B, Yang H, Wu J, Wang J, Ru W, Cheng J, et al. CircSVIL regulates bovine myoblast development by inhibiting STAT1 phosphorylation. Sci China Life Sci. 2021. 10.1007/s11427-020-1908-2. 10.1007/s11427-020-1908-2 34024027

[52] Ju X, Liu Y, Shan Y, Ji G, Zhang M, Tu Y, Zou J, Chen X, Geng Z, Shu J (2021). Analysis of potential regulatory LncRNAs and CircRNAs in the oxidative myofiber and glycolytic myofiber of chickens. Sci Rep.

[53] Lin J, Wu H, Tarr PT, Zhang CY, Wu Z, Boss O, Michael LF, Puigserver P, Isotani E, Olson EN, Lowell BB, Bassel-Duby R, Spiegelman BM (2002). Transcriptional co-activator PGC-1 alpha drives the formation of slow-twitch muscle fibres. Nature..

[54] Ventura-Clapier R, Garnier A, Veksler V (2008). Transcriptional control of mitochondrial biogenesis: the central role of PGC-1alpha. Cardiovasc Res.

[55] Zhang GM, Guo YX, Deng MT, Wan YJ, Deng KP, Xiao SH, Meng FX, Wang F, Lei ZH (2019). Effect of PPARGC1A on the development and metabolism of early rabbit embryos in vitro. Mol Reprod Dev.

