# Supplementary material for: circPTPN4 regulates myogenesis via the miR-499-3p/NAMPT axis
Source: J Anim Sci Biotechnol. 2022 Feb 14;13:2. doi: 10.1186/s40104-021-00664-1 (PMC8842800; doi:10.1186/s40104-021-00664-1)
Supplement: Supplementary file 1 — Additional file 1. [file 40104_2021_664_MOESM1_ESM.zip › Supplementary Material.docx]

**Table S1. Information of Primers.**

| **Primer name** | **Primer sequences, 5’ to 3’** | **Annealing temperature, ◦C** | **Usage** |
| --- | --- | --- | --- |
| circPTPN4-convergent | F: CGCACGTTTCCGATTGCC | 56 | circRNA verification |
|  | R: ATCCAGGAGAAGAATGTTGC |  |  |
| circPTPN4-divergent | F: TGGAGTGATGTCAGGTGGAA | 56 | circRNA verification |
|  | R: AGCAGGCAATCGGAAACG |  |  |
| qPCR-circPTPN4 | F: GAGTGATGTCAGGTGGAA | 54 | qPCR |
|  | R: CAGCTCAGATGCTCGTAC |  |  |
| qPCR-PTPN4 | F: ATCTGTGGAAAGCCTGTG | 58 | qPCR |
|  | R: GTGGAGACCGTGATGGAA |  |  |
| qPCR-FOXA2 | F: ACCGAGGGAAAAGGTCG | 50 | qPCR |
|  | R: CCCCGTAGTAGTTGCTCCA |  |  |
| qPCR-NAMPT | F: AAAGAAGTGTATAGGGAGCA | 58 | qPCR |
|  | R: ATTTGTGAGCCAGTAGCA |  |  |
| qPCR-CDKN1A | F: CCCGTAGACCACGAGCAGAT | 60 | qPCR |
|  | R: CGTCTCGGTCTCGAAGTTGA |  |  |
| qPCR-CDKN1B | F: TCGCTGTGCTGGGCTGAA | 56 | qPCR |
|  | R: CAAGGACGAAAGGATGTGGG |  |  |
| qPCR-PCNA | F: GTGCTGGGACCTGGGTT | 56 | qPCR |
|  | R: CGTATCCGCATTGTCTTCT |  |  |
| qPCR-MyHC | F: CTCCTCACGCTTTGGTAA | 53 | qPCR |
|  | R: TGATAGTCGTATGGGTTGGT |  |  |
| qPCR-MYOD | F: GCTACTACACGGAATCACCAAAT | 53 | qPCR |
|  | R: CTGGGCTCCACTGTCACTCA |  |  |
| qPCR-MYOG | F: CGGAGGCTGAAGAAGGTGAA | 53 | qPCR |
|  | R: CGGTCCTCTGCCTGGTCAT |  |  |
| qPCR-COX2 | F: GTAGATGCCCAAGAAGTT | 54 | qPCR |
|  | R: GTTTGATTTAGTCGTCCAG |  |  |
| qPCR-β-globin | F: CAGCCAGGTGGAGGATTT | 58 | qPCR |
|  | R: GAATAGGAGGACCCTCTGTTAG |  |  |
| qPCR-GPI | F: ATTCACTTTGGGAGCAATC | 54 | qPCR |
|  | R: ACTCCAACTCTGGCTCAAT |  |  |
| qPCR-HK1 | F: CTGGATCTCGGTGGTTCTTAC | 54 | qPCR |
|  | R: TTGTCGGCACGGGAAAGA |  |  |
| qPCR-PGAM1 | F: GCGAGGCTCAGGTGAAGAT | 54 | qPCR |
|  | R: GTCCTCCGTCAGGTCAGC |  |  |
| qPCR-PGK1 | F: CCCTGGATAAGGTGGATG | 54 | qPCR |
|  | R: TTGTCAGGCATGGGAACT |  |  |
| qPCR-PYGL | F: ACATTTGCCTACACGAACC | 54 | qPCR |
|  | R: TGCCTCCCTCCTCTATCA |  |  |
| qPCR-SOX6 | F: TCAGGTTCAGGGTCACATGCC | 57 | qPCR |
|  | R: TTGCTGGAGCTGTAAAGGGC |  |  |
| qPCR-TNNC1 | F: GTTGAGCAGTTGACAGAAGA | 57 | qPCR |
|  | R: GAACCATCATAACAAGGAAC |  |  |
| qPCR-TNNC2 | F: GAGCAGCAAAGATGGCGTCA | 57 | qPCR |
|  | R: ATCACCGTGCCCAACTCCTT |  |  |
| qPCR-TNNI1 | F: GAGGAGTGGGAGCAGGAGAT | 57 | qPCR |
|  | R: TTCGTCCACAATCTCAACCT |  |  |
| qPCR-TNNT1 | F: GAGCCGCACGGAGAAGGAGC | 57 | qPCR |
|  | R: CCCGAAGTGGGGCATGTTGG |  |  |
| qPCR-TNNT3 | F: AGAGGGAAGAAGCAAACAGC | 57 | qPCR |
|  | R: GTCCCACAGTTCCTTAGCCT |  |  |
| qPCR-β-actin | F: GATATTGCTGCGCTCGTTG | 56 | qPCR |
|  | R: TTCAGGGTCAGGATACCTCTTT |  |  |
| pGL3-PTPN4-WT | F: **CTCGAG**TCTGAGACTTCACTGAAGCAG | 60 | Vector construction |
|  | R: **AAGCTT**AATGACATTTACAGTTCAAATA |  |  |
| pGL3-PTPN4-MT | F: TCTGATCCTCGTTTTATCCCACTGGTGTAGAACCTCCAAGAC | 60 | Vector construction |
|  | R: GTCTTGGAGGTTCTACACCAGTGGGATAAAACGAGGATCAGA |  |  |
| pcDNA3.1-FOXA2-FLAG | F: **AAGCTT**ATGCTGGGAGCGGTGAAA | 60 | Vector construction |
|  | R: **CTCGAG**GGAGGAGTTCATGATGGGCC |  |  |
| pcDNA3.1-FOXA2 | F: **AAGCTT**ATGCTGGGAGCGGTGAAA | 60 | Vector construction |
|  | R: **CTCGAG**TTAGGAGGAGTTCATGATGGGC |  |  |
| pcD25-circPTPN4 | F: **GAATTC**TAATACTTTCAGACCCTGTGTGGACAGTAATGAC | 60 | Vector construction |
|  | R: **GGATCC**AGTTGTTCTTACCAAGGAAAGGTGTTAATTCGG |  |  |
| pmirGLO-circPTPN4-WT | F: **CTCGAG**TACATCAGCAGCACATAG | 60 | Vector construction |
|  | R: **GTCGAC**TGTTAATTCGGACTCTGT |  |  |
| pmirGLO-circPTPN4-MT | F: ACAATGAAATAATGATTGGATGTCGTGCAGGTGGAATACTGA | 60 | Vector construction |
|  | R: TCAGTATTCCACCTGCACGACATCCAATCATTATTTCATTGT |  |  |
| pmirGLO-NAMPT-WT | F: **CTCGAG**CTAGGGTTTGTGGTGAAGA | 60 | Vector construction |
|  | R: **GTCGAC**GCTCTGTACTATTTACCTGCT |  |  |
| pmirGLO-NAMPT-MT | F: GTATGTAATTATACATTTAATGTCGTGATTGTTTCAAAAATA | 60 | Vector construction |
|  | R: TATTTTTGAAACAATCACGACATTAAATGTATAATTACATAC |  |  |

Sequences in bold represent the enzyme cutting sites.

**Table S2. Oligonucleotide sequences in this study.**

| **Fragment name** | **Sequences, 5’ to 3’** |
| --- | --- |
| si-circPTPN4 | CACCTTTCCTTGACCCTGT |
| miR-499-3p mimic | AACAUCACUUUAAGUCUGUGCU |

**Table S3. Differential expression analysis of circRNAs between pectoralis major and soleus in 7-week-old Xinghua chicken.**

Separate Excel file.

**Table S4. The full-length sequence of *circPTPN4*.**

ACCCTGTGTGGACAGTAATGACCGCACGTTTCCGATTGCCTGCTGGCAGAACCTACAATGTACGAGCATCTGAGCTGGCACGAGACAGGCAGCATACGGAGGTGGTCTGCAACATTCTTCTCCTGGATAACACTGTACAAGCTTTCAGAGTTAATAAACACGATCAGGGACAAGTTCTGTTGGATATAGTCTTCAAACATCTCGATTTGACAGAGAGAGATTACTTTGGTTTACAGCTGGCGGATGAATCCACTGATAACCCTAGGTGGTTGGATCCAAACAAACCTATCAGGAAACAATTAAAAAGAGGATCTCCACACAGTTTGAACTTGAGAGTTAAGTTTTTTGTAAGTGACCCAAACAAGCTCCAAGAAGAATATACAAGGTACCAGTATTTTTTACAAATTAAACAGGACATTCTTACTGGAAGATTGCCCTGTCCATATAATACTGCTGCTCTTCTGGCTTCCTATGCTGTTCAATCCGAGCTGGGAGACTACAACCATTTGGAAAACCTGCCAGGCTACCTCTCAGACTATTCTTTCATCCCTGGCCAGCCTCAAGACTTTGAAAAAGAGATAGCAAAGTTACATCAGCAGCACATAGGGTTGTCTCCTGCAGAAGCGGAGTTTAATTATCTCAATACAGCACGTACCTTAGAACTCTATGGAGTTGAATTGCACTATGCAAGGGATCAGAGTAACAATGAAATAATGATTGGAGTGATGTCAGGTGGAATACTGATCTTTAAGAACAGAGTCCGAATTAACACCTTTCCTTG


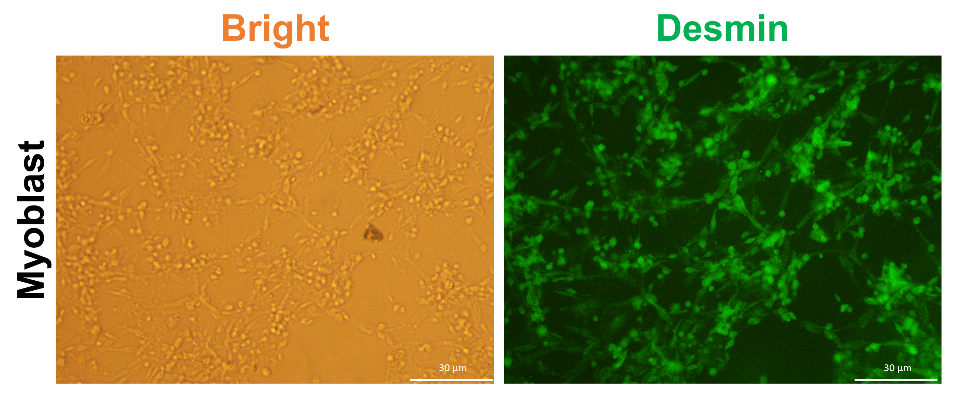


**Figure S1.** **Identification of the purity of isolated primary myoblasts.** Left. Microscopic image of isolated primary myoblasts. Right. Desmin immunostaining of isolated primary myoblasts.


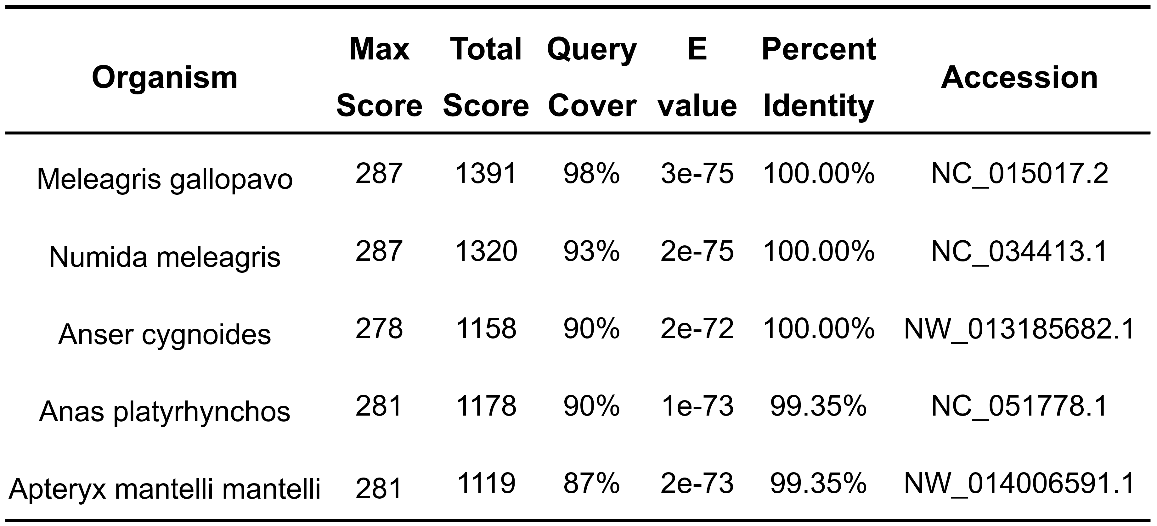


**Figure S2. Nucleotide sequence conservative analysis of *circPTPN4* was performed by using the NCBI’s BLAST.** A total of twelve species, including *Anas platyrhynchos*, *Anser cygnoides*, *Apteryx mantelli mantelli*, *Aquila chrysaetos*, *Coturnix japonica*, *Geospiza fortis*, *Homo sapiens*, *Meleagris gallopavo*, *Melopsittacus undulatus*, *Mus musculus*, *Numida meleagris*, and *Zebra finch* were used for Nucleotide BLAST. The top 5 most conservative results were listed above.


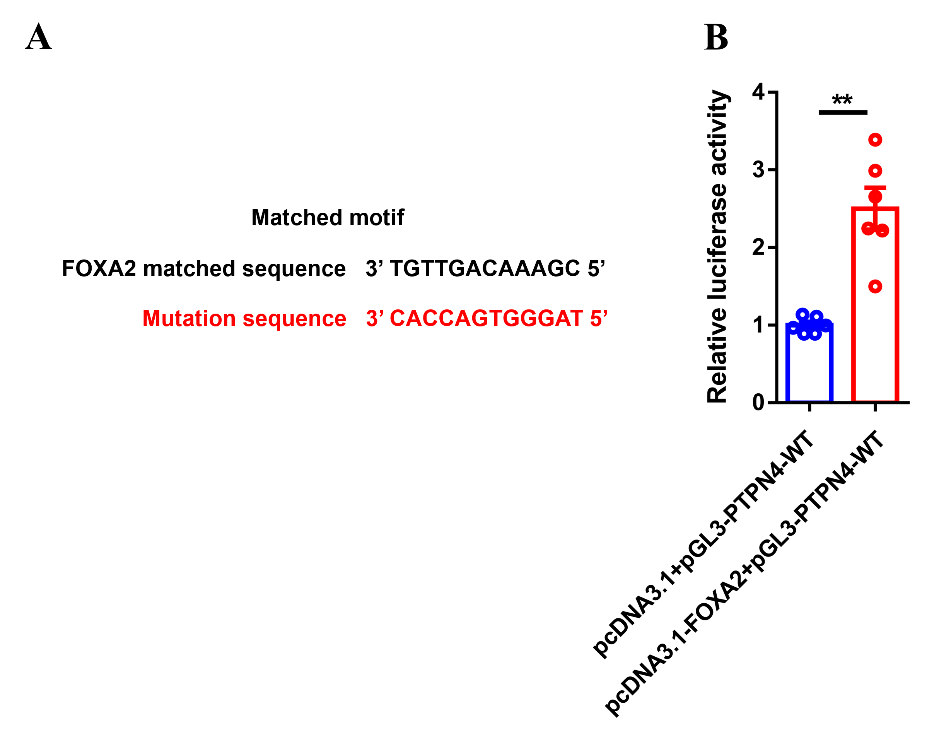


**Figure S3. FOXA2 regulates the promoter activity of *PTPN4*.** (**A**) The potential binding site of FOXA2 in *PTPN4* promoter region. The mutant sequence of FOXA2 binding site is highlighted in red. (**B**) The transcriptional activity of *PTPN4* core promoter region after *FOXA2* overexpression. Results are shown as mean ± SEM. In panel (**B**), the statistical significance of differences between means was assessed using independent sample *t*-test. (***P* < 0.01).

**
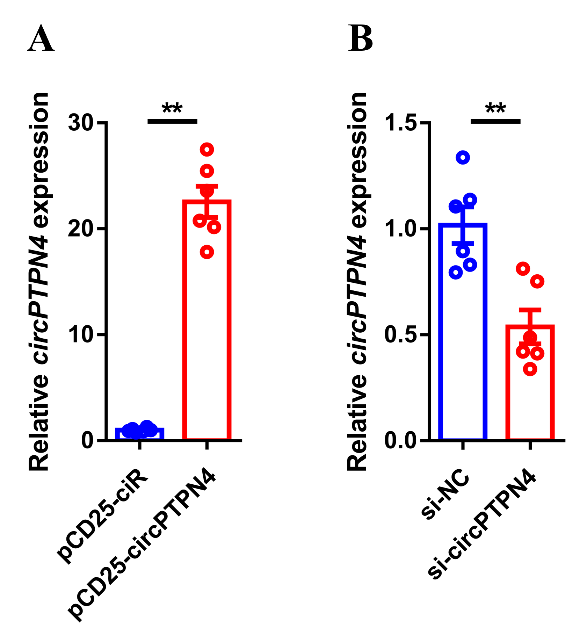
**

**Figure S4. Analysis of overexpression and interference efficiency of *circPTPN4* in CPMs.** (**A**) Relative *circPTPN4* expression with *circPTPN4* overexpression in CPMs. (**B**) Relative *circPTPN4* expression with *circPTPN4* interference in CPMs. Results are presented as mean ± SEM. In all panels, the statistical significance of differences between means was assessed using independent sample *t*-test. (***P* < 0.01).


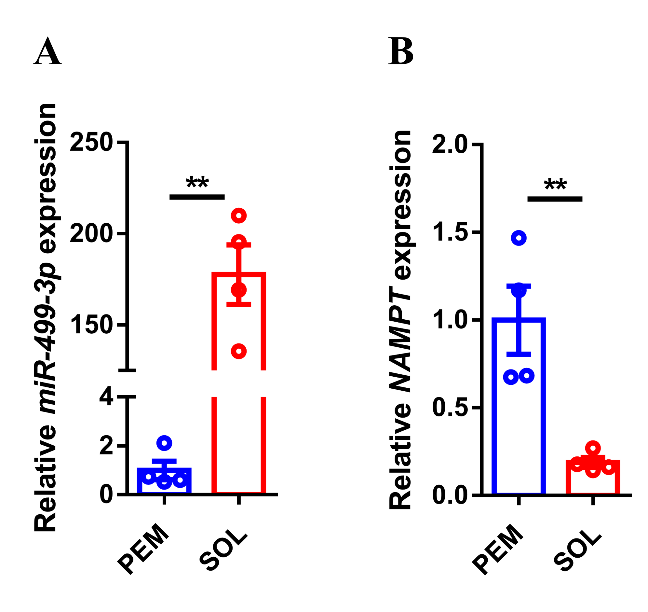


**Figure S5. Relative *miR-499-3p* and *NAMPT* expression in pectoralis major (PEM) and soleus (SOL) of 7-week-old Xinghua chicken.** (**A**) Relative *miR-499-3p* expression in PEM and SOL of 7-week-old Xinghua chicken. (**B**) Relative *NAMPT* expression in PEM and SOL of 7-week-old Xinghua chicken. Results are presented as mean ± SEM. In all panels, the statistical significance of differences between means was assessed using paired *t*-tests. (***P* < 0.01).
