# Supplementary figures and images for: circPTPN4 regulates myogenesis via the miR-499-3p/NAMPT axis
Source: J Anim Sci Biotechnol. 2022 Feb 14;13:2. doi: 10.1186/s40104-021-00664-1 (PMC8842800; doi:10.1186/s40104-021-00664-1)

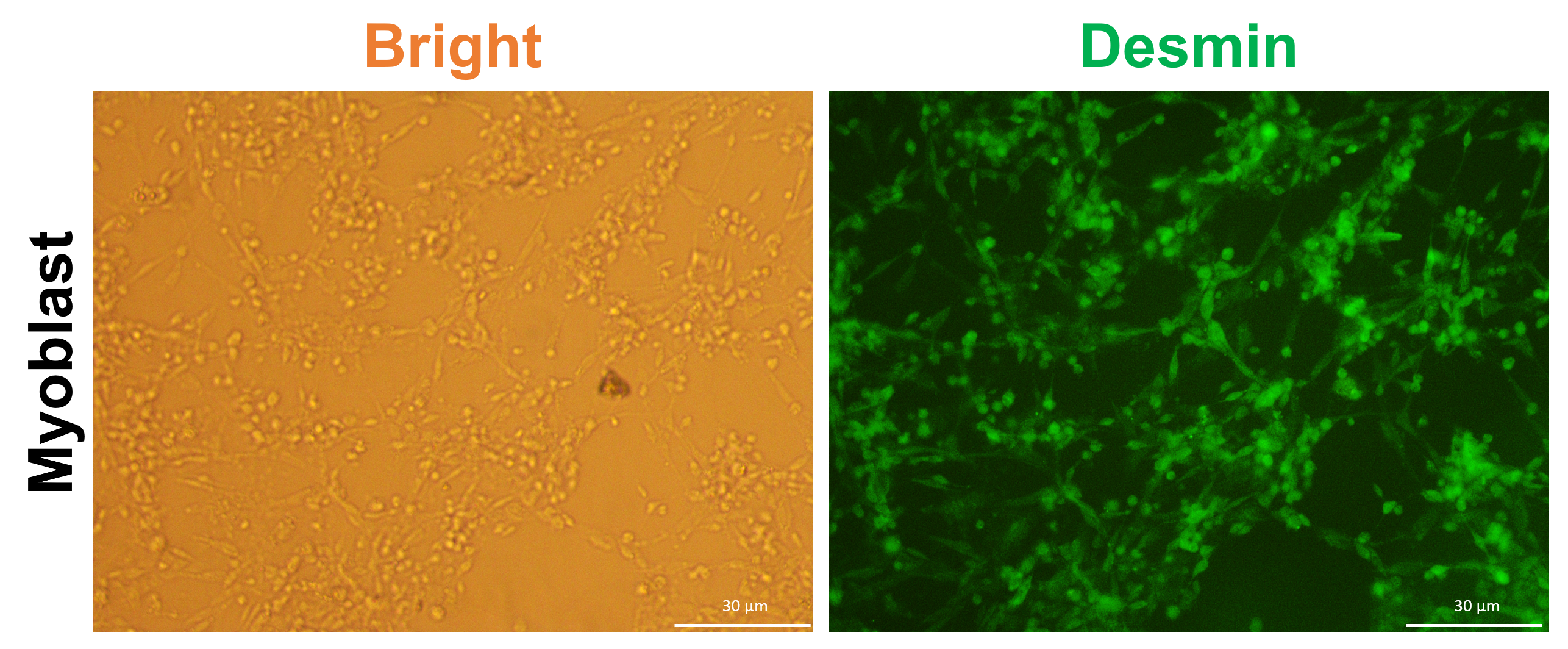

Supplement: Supplementary file 1 — Additional file 1. [file 40104_2021_664_MOESM1_ESM.zip › Figure S1.tif]

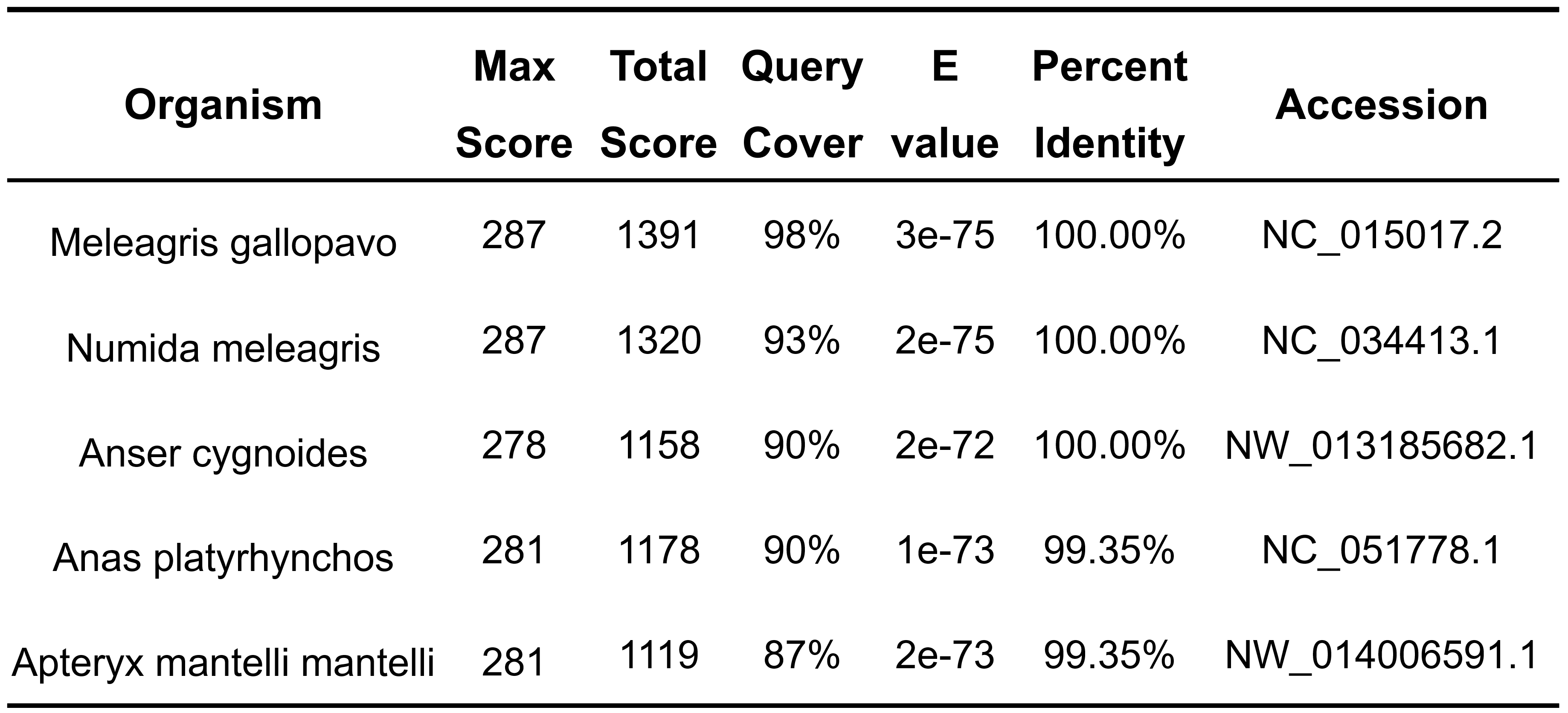

Supplement: Supplementary file 1 — Additional file 1. [file 40104_2021_664_MOESM1_ESM.zip › Figure S2.tif]

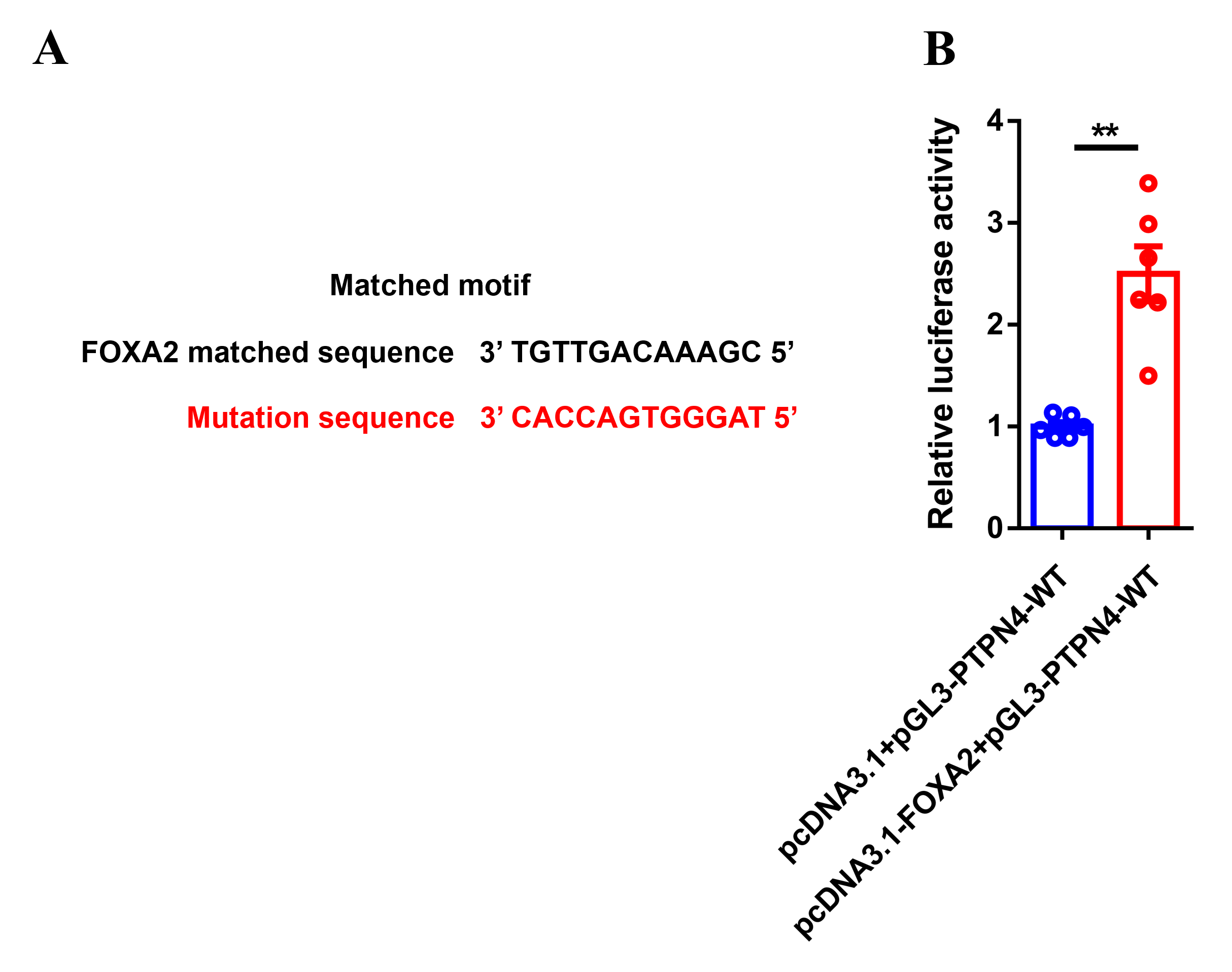

Supplement: Supplementary file 1 — Additional file 1. [file 40104_2021_664_MOESM1_ESM.zip › Figure S3.tif]

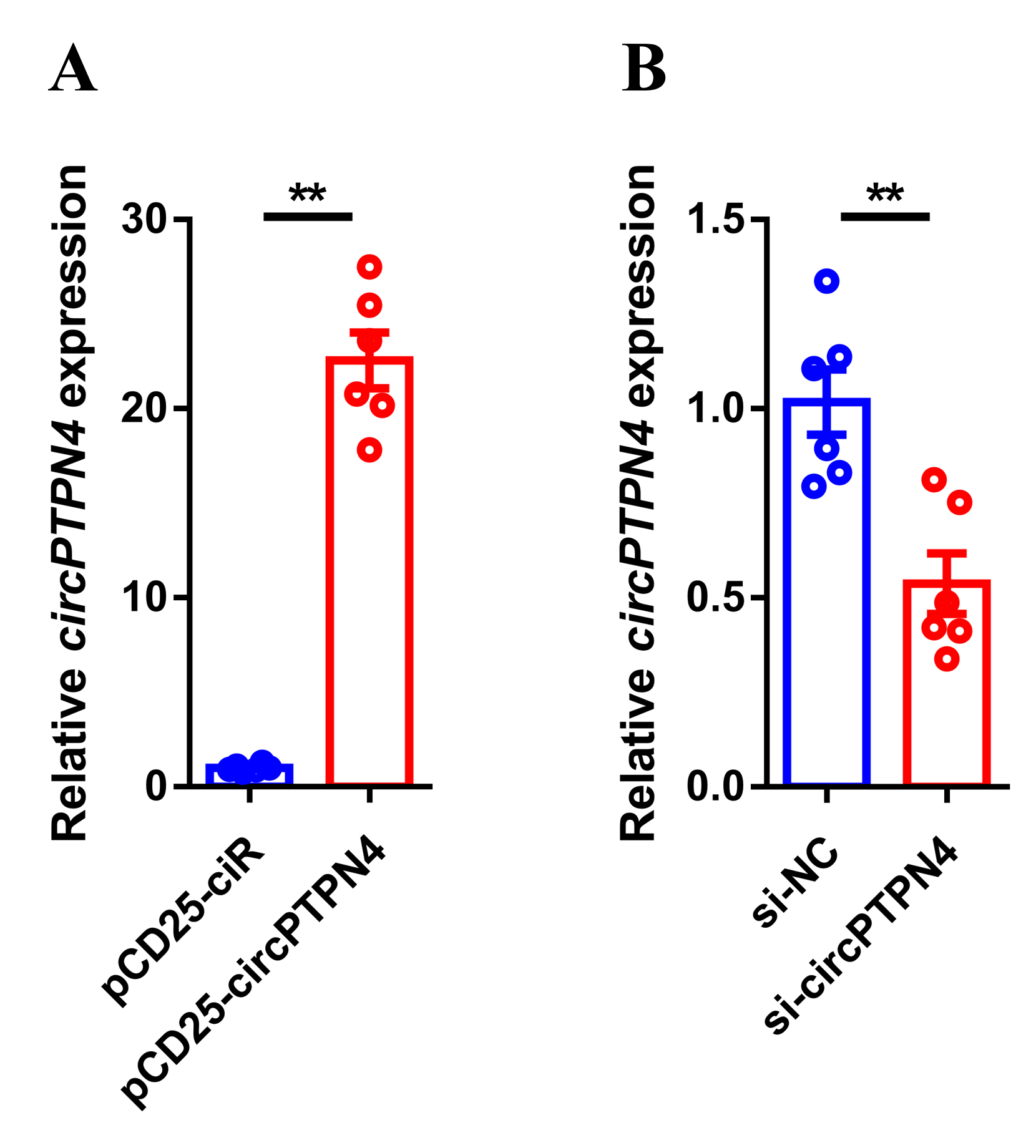

Supplement: Supplementary file 1 — Additional file 1. [file 40104_2021_664_MOESM1_ESM.zip › Figure S4.tif]

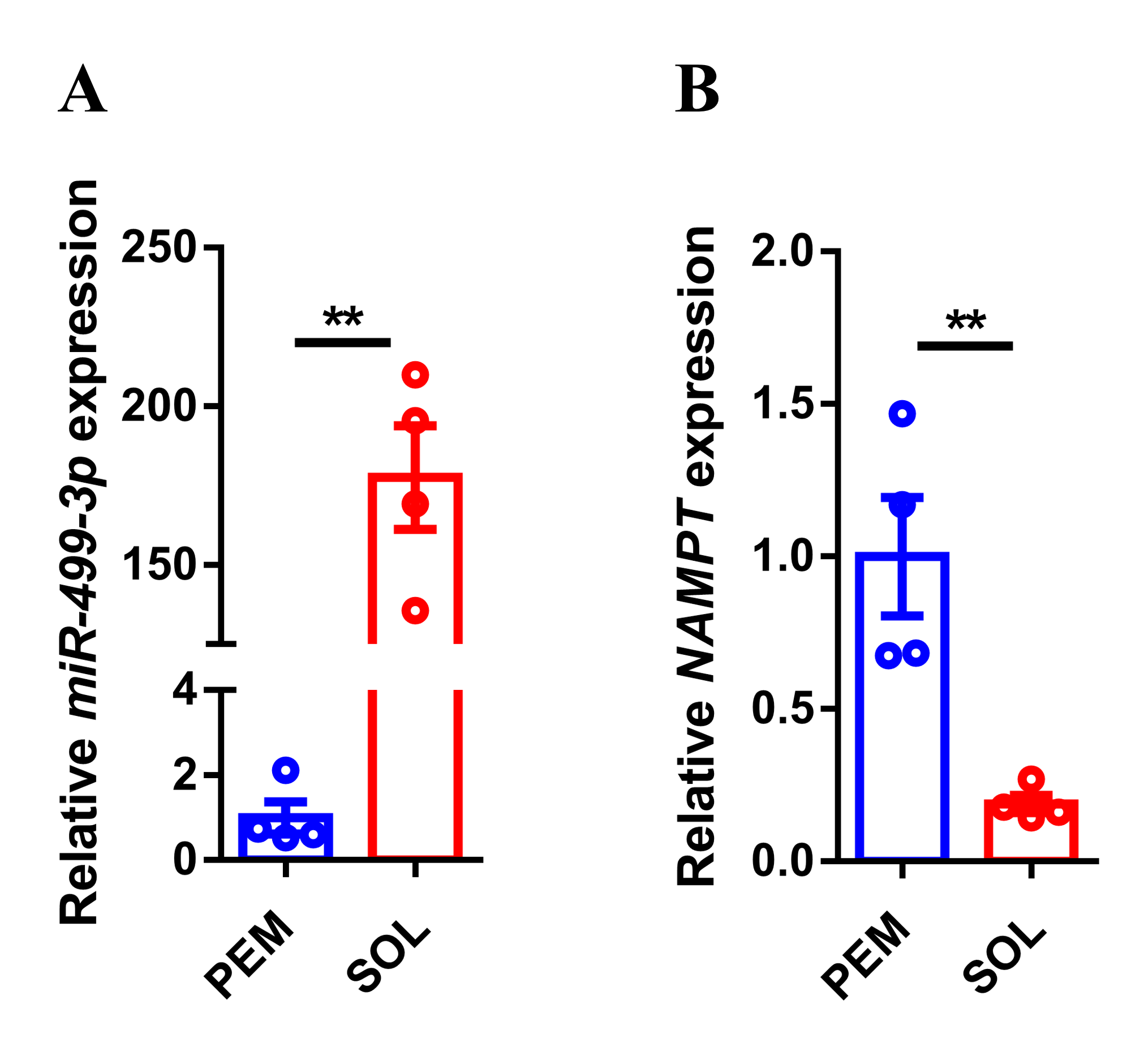

Supplement: Supplementary file 1 — Additional file 1. [file 40104_2021_664_MOESM1_ESM.zip › Figure S5.tif]
